# Cytokine Signatures Outperform Immune Subsets in Machine Learning Models for Predicting Acute Graft‐Versus‐Host Disease at Neutrophil Engraftment

**DOI:** 10.1155/jimr/1066614

**Published:** 2026-02-23

**Authors:** Mohini Mendiratta, Praful Pandey, Shobhit Pandey, Sandeep Rai, Meenakshi Mendiratta, Hridayesh Prakash, Shuvadeep Ganguly, Archana Sasi, Ritu Gupta, Prabhat Singh Malik, Raja Pramanik, Sachin Kumar, Baibaswata Nayak, Riyaz Ahmed Mir, Sameer Bakhshi, Deepam Pushpam, Mukul Aggarwal, Aditya Kumar Gupta, Rishi Dhawan, Tulika Seth, Manoranjan Mahapatra, Ranjit Kumar Sahoo

**Affiliations:** ^1^ Department of Medical Oncology, Dr. B. R. Ambedkar Institute Rotary Cancer Hospital, All India Institute of Medical Sciences, New Delhi, Delhi, India, aiims.edu; ^2^ Indraprastha Institute of Information Technology, New Delhi, Delhi, India, iiitd.ac.in; ^3^ Laboratory Oncology Unit, Dr. B. R. Ambedkar Institute Rotary Cancer Hospital, All India Institute of Medical Sciences, New Delhi, Delhi, India, aiims.edu; ^4^ Stem Cell Facility (DBT-Centre of Excellence for Stem Cell Research), All India Institute of Medical Sciences, New Delhi, Delhi, India, aiims.edu; ^5^ Amity Centre for Translational Research, Amity University, Sector 125, Noida, Uttar Pradesh, India, amity.edu; ^6^ Department of Gastroenterology and Human Nutrition, All India Institute of Medical Sciences, New Delhi, Delhi, India, aiims.edu; ^7^ Department of Biochemistry, All India Institute of Medical Sciences, New Delhi, Delhi, India, aiims.edu; ^8^ Department of Hematology, All India Institute of Medical Sciences, New Delhi, Delhi, India, aiims.edu; ^9^ Department of Pediatrics Oncology, All India Institute of Medical Sciences, New Delhi, Delhi, India, aiims.edu

**Keywords:** aGvHD, allogeneic hematopoietic stem cell transplantation, cytokines, engraftment, immunophenotyping, machine learning

## Abstract

**Background:**

Acute graft‐versus‐host disease (aGvHD) is a major immune complication of allogeneic hematopoietic stem cell transplantation (Allo‐HSCT), driven by complex immune‐cytokine interactions. This study employed machine learning (ML) algorithms to develop early predictive models for aGvHD using immune and cytokine profiles of Allo‐HSCT recipients at the time of engraftment.

**Materials and Methods:**

Seventy patients with hematological disorders undergoing their first Allo‐HSCT were recruited prospectively. Peripheral blood immune subsets and cytokines were analyzed using flow cytometry and ELISA, respectively. ML models, including support vector classifier (SVC), decision tree, and random forest, were trained on 48 features: 34 immune subsets and 14 cytokines.

**Results:**

Patients who developed aGvHD exhibited a reduced CD4^+^/CD8^+^ ratio, lower Tregs, elevated Th1, Th17, cytotoxic natural killer (NK) cells, dendritic cells (DCs), B cell, and increased proinflammatory cytokines (IFN‐γ, IL‐1β, IP‐10, TNF‐α, IL‐17α, IL‐12p70, MIP‐1α, MIP‐1β, and RANTES). ML models demonstrated excellent predictive performance, with cytokine profiles alone or combined with immune data achieving perfect accuracy (1.00), followed by T‐cell (0.96), NK cell (0.93), DC (0.90), and B cell (0.86) models.

**Conclusion:**

Cytokine profiles emerged as superior predictors over immune subsets, supporting their integration into ML‐based aGvHD risk prediction. These findings provide a foundation for developing biomarker‐guided strategies for early aGvHD detection and management.

## 1. Introduction

Acute graft‐versus‐host disease (aGvHD) remains a major complication following allogeneic hematopoietic stem cell transplantation (Allo‐HSCT), significantly contributing to morbidity and mortality. The ability to predict aGvHD before clinical onset could transform posttransplant management by enabling early intervention and personalized immunomodulation, which is an unmet need.

Neutrophil engraftment [1] represents a biologically and clinically meaningful time point for immune monitoring, as it coincides with the initial reestablishment of donor hematopoiesis and the onset of critical immune interactions that may drive alloimmunity. Early immune recovery during this period offers a window to detect subclinical immune dysregulation predictive of aGvHD.

In this study, we performed high‐dimensional immune profiling at the time of neutrophil engraftment in Allo‐HSCT recipients to explore its association with subsequent aGvHD development. By integrating cellular immune signatures and soluble cytokine profiles [2, 3] using machine learning (ML) approaches, we aimed to identify early, predictive biomarkers of aGvHD and to better understand the immunological mechanisms. Our findings provide novel insights into the early immunological landscape postengraftment and propose candidate markers for risk stratification and potential therapeutic targeting. In addition, we investigated the kinetics of immune cell reconstitution in patients with and without aGvHD to identify distinct immunological trajectories. To our knowledge, this is the first study to comprehensively profile immune cell subsets and cytokines specifically at neutrophil engraftment in Allo‐HSCT recipients while applying ML techniques to develop predictive models for aGvHD. A version of this manuscript was previously made publicly available as a preprint on bioRxiv [4].

## 2. Materials and Methods

### 2.1. Sex as a Biological Variable

Our study involved both male and female human subjects. Efforts were made to recruit participants of both sexes, and data were analyzed without prior assumptions regarding sex‐based differences. However, the sample size was not powered to detect sex‐specific effects. Therefore, while the findings are expected to be broadly relevant across sexes, potential sex‐dimorphic responses cannot be ruled out.

### 2.2. Ethical Approval

The study involved human subjects, and ethical approval was obtained from the Institutional Ethics Committee at the All India Institute of Medical Sciences (AIIMS), New Delhi, India (Ref. No.: IECPG‐542/23.09.2020). Informed written consent was obtained from the participants, and all procedures followed the guidelines and regulations approved by the ethics committee.

### 2.3. Study Population

Patients diagnosed with hematological diseases who underwent their first Allo‐HSCT were enrolled in this study. Patients with a known history of viral infections (HIV, HBV, and HCV), immunodeficiency disorders, or autoimmune diseases were excluded from the study.

A total of 70 patients were recruited from the Department of Medical Oncology, Hematology, and Paediatric Oncology at the AIIMS, New Delhi, between September 2020 and July 2023 after obtaining approval. The sample size was determined based on a priori power calculation using the historical incidence of aGvHD observed at our institute. Assuming an expected aGvHD incidence of ~35%–40%, with a desired power of 80% and a significance level (*α*) of 0.05, the calculated sample size required to detect significant differences in immune parameters between aGvHD and non‐aGvHD patients was ~64. To account for potential dropouts or missing data, the final recruitment target was set at 70 patients. This sample size was considered adequate for exploratory analysis and development of predictive models for aGvHD based on immune profiling.

Peripheral blood samples were collected at predefined posttransplant time points on days +14, +30, +60, +100, and +180. These time points were chosen to align with clinically relevant phases of immune reconstitution and GvHD evolution following Allo‐HSCT. Day +14 reflects the early engraftment period characterized by innate immune recovery and establishment of the initial inflammatory milieu. Day +30 represents the early postengraftment phase during which acute GvHD commonly manifests and adaptive immune activation accelerates. Days +60 and +100 represent the peak window for acute GvHD and allow assessment of dynamic changes in immune subsets as the system begins transitioning toward stabilization. Day +180 corresponds to the late posttransplant phase and allows longer‐term immune reconstitution and assessment of regulatory recovery and the emergence of chronic immune alterations. This sampling framework is consistent with the routine clinical monitoring schedule in transplant centers and was chosen to comprehensively capture early perturbations and sustained immunologic adaptation after transplantation.

A control group comprising age‐ and sex‐matched healthy individuals (*n* = 20) was also included. For these healthy volunteers, a single PB sample was collected at the time of enrollment. While they served as baseline comparators, their sampling strategy was limited to this one‐time collection.

### 2.4. Enumeration of Immune Cellular Subsets

White blood cells (WBCs) were isolated from PB using the bulk lysis method. Briefly, the PB sample was diluted with 1× RBC lysis buffer (Invitrogen, Thermo Fisher Scientific, USA) in a 1:3 ratio and incubated for 15 min at room temperature. Following incubation, the diluted blood was centrifuged at 2000 rpm for 5 min, and the pellet was washed with 1× PBS (Thermo Fisher Scientific, USA) at 2000 rpm for 5 min. The resulting pellet was resuspended in 200 μL 1× PBS (Thermo Fisher Scientific, USA), and the cells were stained with fluorochrome‐conjugated antihuman monoclonal antibodies against surface markers including CD27, CD20, CD19, CD141, KIR, CD16, CD1c, CD56, CD11c, CD123, CD7, HLA‐DR, CD3, CD45RA, CD25, CD4, CD8 (Beckman Coulter, USA), and IgD (Thermo Fisher Scientific, USA) for 40 min in the dark at room temperature. Fluorochrome‐conjugated antihuman monoclonal antibodies for chemokine receptors—CCR7, CXCR3, CCR10, CCR6, CCR4, and CXCR4 (Becton Dickinson, USA)—were used to analyze subtypes of helper T‐cell, cytotoxic T‐cell, and effector memory helper T‐cell, and their staining was performed at 37°C, mimicing physiological conditions that help to maintain the receptors in a more accessible state for antibody binding [5, 6]. After surface staining, cells were fixed and permeabilized using BD Pharmingen Transcription Factor Buffer Set (Becton Dickinson, USA), followed by staining with antihuman monoclonal antibodies targeting cytoplasmic markers such as FOXP3 (Becton Dickinson, USA) for 30 min at room temperature in the dark. A minimum of 5,000,000 events were acquired using the DxFlex flow cytometer (Beckman Coulter, USA), and the data were analyzed using the Kaluza software (Beckman Coulter, USA). A representation of immune cells and their subtypes, with profiling markers (mentioned in italics) used for immunophenotyping, is depicted in Figure S1.

### 2.5. Cytokines Profiling of Allo‐HSCT Recipients

Cytokine levels, including IFN‐γ, IL‐10, MIP‐1α, IL‐1β, TNF‐α, and IL‐17α, were quantified using ELISA kits (Thermo Fisher Scientific, USA) according to the manufacturer’s instructions. Briefly, PB samples were obtained from three cohorts: healthy controls, non‐aGvHD patients, and aGvHD patients, and the measurements in the patient cohorts were performed at the time of neutrophil engraftment, defined as an absolute neutrophil count (ANC) exceeding 500/μL. PB samples were collected in a clot activator tube, allowed to clot at room temperature for 30 min, and then centrifuged at 3500 rpm for 10 min to obtain serum. Samples were aliquoted and stored at −80°C until analysis. Before assay, samples were thawed on ice and brought to room temperature. All standards, controls, and serum samples were run in triplicate. Absorbance was measured using a PR4100 microplate reader (Bio‐Rad, USA). A standard curve was generated using recombinant cytokine standards, and concentrations in samples were calculated using curve‐fitting software provided by the plate reader manufacturer.

### 2.6. ML Algorithm for Predicting aGvHD

To identify early immunological predictors of aGvHD, ML models were employed using immune profiling data. The models included support vector classifier (SVC) with a radial basis function (RBF) kernel, decision tree, and random forest algorithms. These models were selected based on their individual strengths. The SVC with an RBF kernel is capable of capturing complex, nonlinear relationships within biological datasets, while decision tree and random forest models offer interpretability and are particularly effective when dealing with heterogeneous data and small‐to‐medium sample sizes.

Before model training, data preprocessing was performed to ensure quality and consistency. Missing values were imputed using median values for continuous variables. All numeric features, including immune cell counts and cytokine levels, were standardized using *z*‐score normalization to facilitate model convergence and to ensure comparability across features. Outliers were identified and capped using the interquartile range (IQR) method. For categorical variables, such as the presence or absence of aGvHD, label encoding was used to convert the data into a machine‐readable format.

To comprehensively evaluate the predictive contribution of different immune parameters, multiple data configurations were created. These included (1) a combined model incorporating both immune cell counts and cytokine levels, (2) models using only immune cell counts, (3) models using only cytokine levels, and (4) subset‐specific configurations, such as T‐cell counts alone, T‐cell and natural killer (NK) cell counts, dendritic cell (DC) counts, and B cell counts. These configurations enabled the isolation of specific immune components and the evaluation of their individual and combined roles in predicting aGvHD onset.

Model performance was assessed using stratified 5‐fold cross‐validation. This approach ensured that each fold preserved the proportion of aGvHD and non‐aGvHD cases, minimizing bias and improving generalizability. For each fold, models were trained on 80% of the data and tested on the remaining 20%, and the results were averaged across folds to obtain robust performance estimates.

### 2.7. Statistical Analysis

All statistical analyses were conducted using GraphPad Prism version 8.4.3. One‐way ANOVA followed by Tukey’s post hoc test was used to compare three or more groups. Data were shown as mean ± S.D., and a *p*‐value of ≤0.05 was considered statistically significant.

## 3. Results

### 3.1. Patient Characteristics

A total of 70 patients who underwent Allo‐HSCT were included in this study. The most common underlying condition was AML, accounting for 35% (*n* = 25) of the cases, followed by aplastic anemia (22.8%, *n* = 16), thalassemia (12.8%, *n* = 9), ALL (11.4%, *n* = 8), MDS (4.28%, *n* = 3), CML‐blast crisis (2.85%, *n* = 2), CLL (1.42%, *n* = 1), and other hematological disorders (8.5%, *n* = 6).

The majority of patients (70%, *n* = 49) underwent MSD transplantation, while 30% (*n* = 21) received haploidentical transplants. 47.14% (*n* = 33) of patients received ATG‐based conditioning, 4.28% (*n* = 3) received PTCy alone, and 1.42% (*n* = 1) received a combination of ATG and PTCy. The remaining 47.14% (*n* = 33) underwent other chemotherapy‐based conditioning regimens. TBI was administered to only one patient (1.42%), whereas the remaining 69 patients (98.58%) received only chemotherapy‐based conditioning for immune ablation.

Grade II–IV aGvHD developed in 25 patients (35.71%), and 67.14% (*n* = 47) of recipients received CNI in combination with MTX, with or without corticosteroids, while 32.85% (*n* = 23) received CNI with MMF, with or without corticosteroids after a median time of *D* + 27 (range: *D* + 16–*D* + 79) after Allo‐HSCT. The study design and patient recruitment flowchart are presented in Figure 1, and detailed clinical characteristics are summarized in Table 1.

Figure 1(A) Schematic representation of study design. (B) Patient recruitment diagram of the study.

**Figure:**
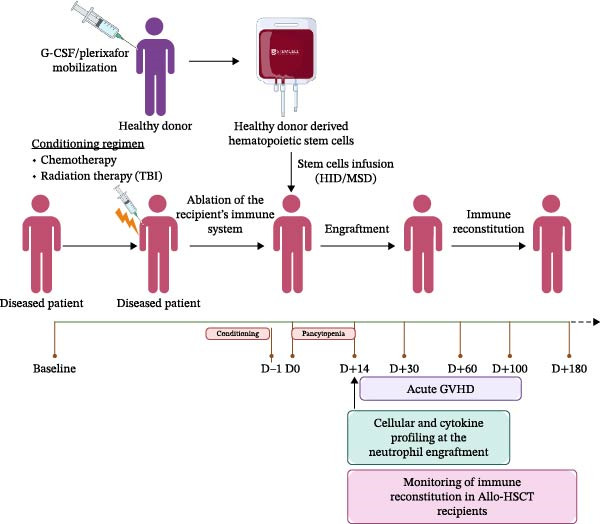
(A)

**Figure:**
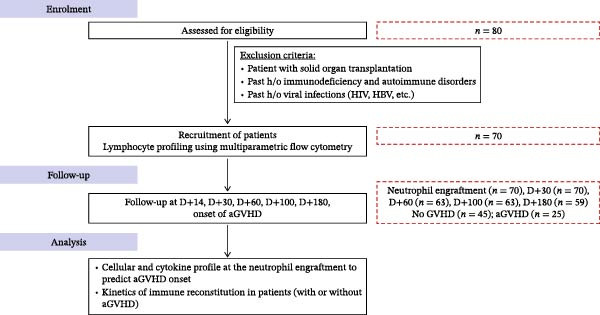
(B)

**Table 1:** Patient characteristics.

Variable	N
Age in years, median patient	21
Sex	
Male	48
Female	22
Diagnosis	
AML	25
ALL	8
CLL	1
CML‐blast crisis	2
Thalassemia	9
Aplastic anemia	16
MDS	3
Others	6
Donor	
Haploidentical	21
Matched sibling donor	49
Conditioning regimen	
ATG only	33
ATG + PTCy	1
PTCy only	3
Others	33
Total body irradiation	
Yes	1
No	69
GVHD (grade II–IV)	25
GVHD prophylaxis	
CNI + MTX ± steroids	47
CNI + MMF ± steroids	23

### 3.2. Aberrant T‐Cell Reconstitution at the Neutrophil Engraftment Contributes to the aGvHD Progression in the Later Phase

T‐cells are well‐established mediators of aGvHD, playing a crucial role in APCs activation during the early phase of disease pathogenesis. In this study, we performed a comprehensive immunophenotypic analysis of T‐cells, their subsets, including Th, Tc, and their respective naïve, effector, effector memory, and central memory populations, and Tregs in Allo‐HSCT recipients at the time of neutrophil engraftment. The gating strategy used for their enumeration is depicted in Figure S1.

During the engraftment phase, all Allo‐HSCT recipients exhibited profound lymphopenia, with significantly reduced absolute lymphocyte counts compared to healthy controls, regardless of aGvHD development. However, patients who later developed aGvHD showed markedly elevated T‐cell counts at engraftment (327.37 vs. 33.33 cells/μL; *p* ≤ 0.0001) (Figure 2A), including both helper (Th: 66.85 vs. 7.76 cells/μL) (Figure 2B) and cytotoxic (Tc: 233.71 vs. 18.21 cells/μL) subsets (*p* ≤ 0.0001 for both) (Figure 2C). Despite the increase in both subsets, the Th/Tc ratio was significantly reduced in aGvHD patients (0.27 vs. 0.45; *p* ≤ 0.0001) (Figure 2D), indicating a skewed cytotoxic T‐cell expansion. These patients also had lower Treg counts (0.030 vs. 0.15 cells/μL; *p* ≤ 0.0001) (Figure 2E), reflecting impaired immune regulation.

Figure 2Profile of T‐cell, Th, Tc, Tregs, and subtypes of Th and Tc cells at the neutrophil engraftment of the patients who either did or did not develop aGvHD in the later phase and healthy control using flow cytometry. Dot plots illustrate the gating strategy for (A) CD3+ T‐cell, (B) CD3+ CD4+ T‐cell, (C) CD3+ CD8+ T‐cell, (D) CD4+/CD8+ T‐cell ratio, (E) Tregs, (F) CCR7+ CD45RA+ Naïve helper T‐cell, (G) CCR7− CD45RA+ effector helper T‐cell, (H) CCR7− CD45RA− effector memory helper T‐cell, (I) CCR7+ CD45RA− central memory helper T‐cell, (J) CCR7+ CD45RA+ Naïve cytotoxic T‐cell, (K) CCR7− CD45RA+ effector cytotoxic T‐cell, (L) CCR7− CD45RA− effector memory cytotoxic T‐cell, and (M) CCR7+ CD45RA− central memory cytotoxic T‐cell. Data are presented as the median with interquartile range for 20 healthy control and 70 patients (aGvHD = 25; non‐aGvHD = 45). Statistical analysis: Mann–Whitney test;  ^∗∗∗∗^≤ 0.0001. aGvHD, acute graft‐versus‐host‐disease; Tc, cytotoxic T‐cell; Th, helper T‐cell; Tregs, regulatory helper T‐cell.

**Figure:**
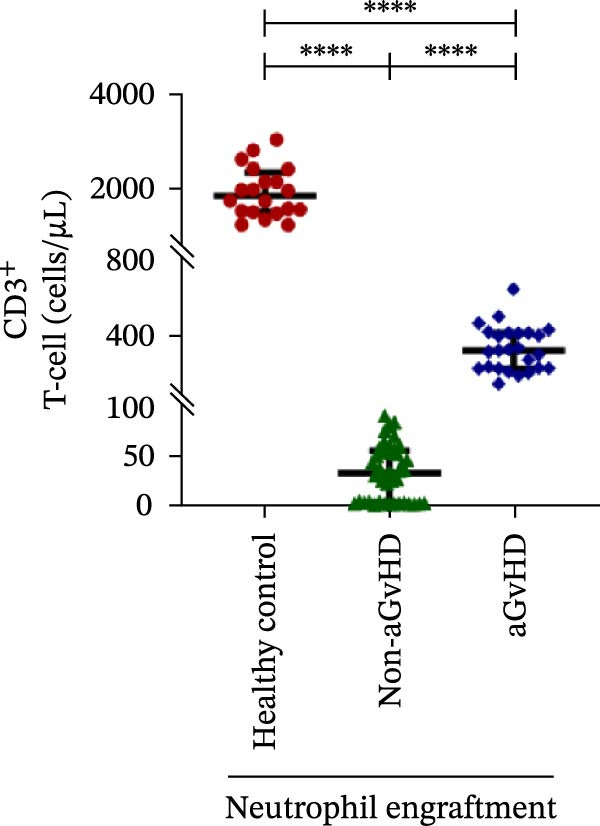
(A)

**Figure:**
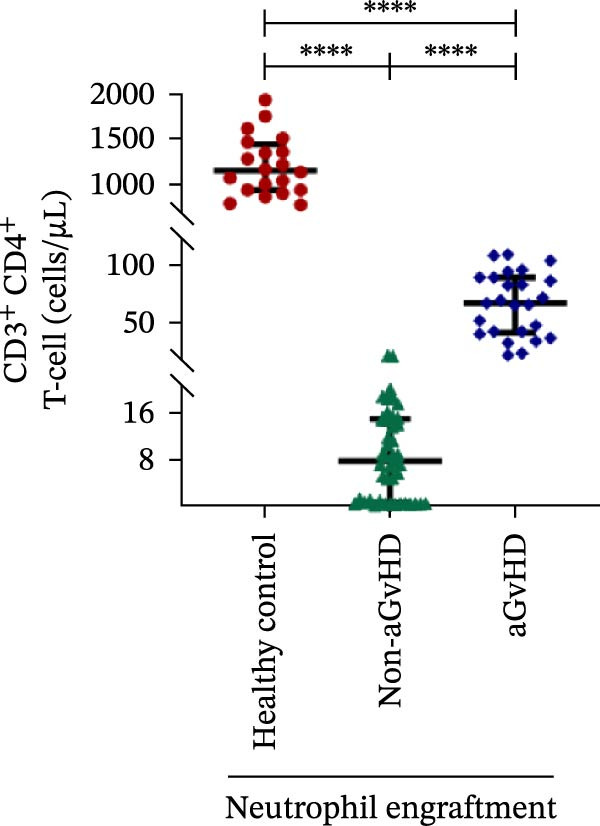
(B)

**Figure:**
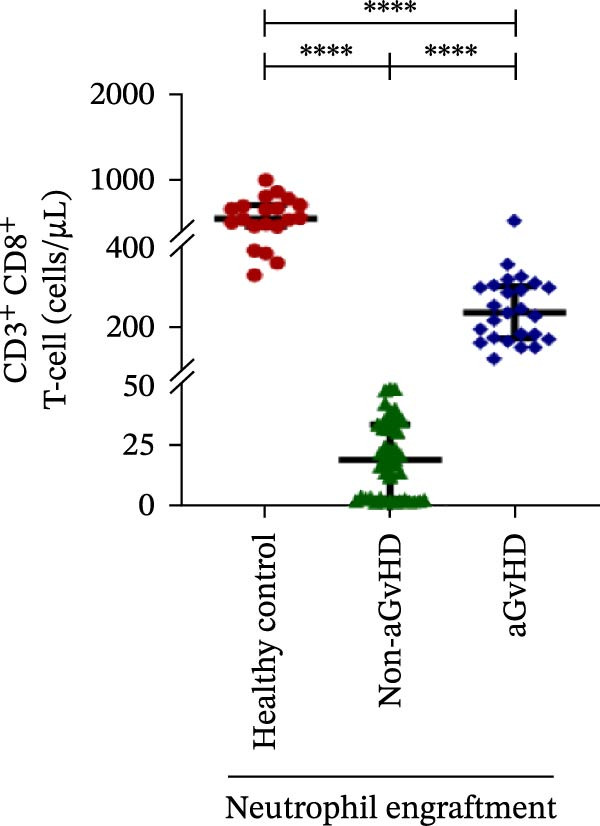
(C)

**Figure:**
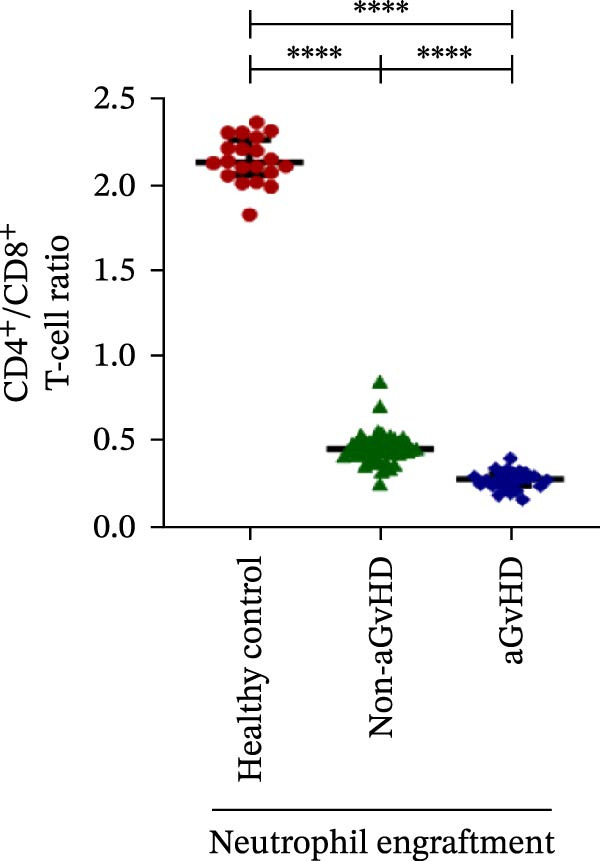
(D)

**Figure:**
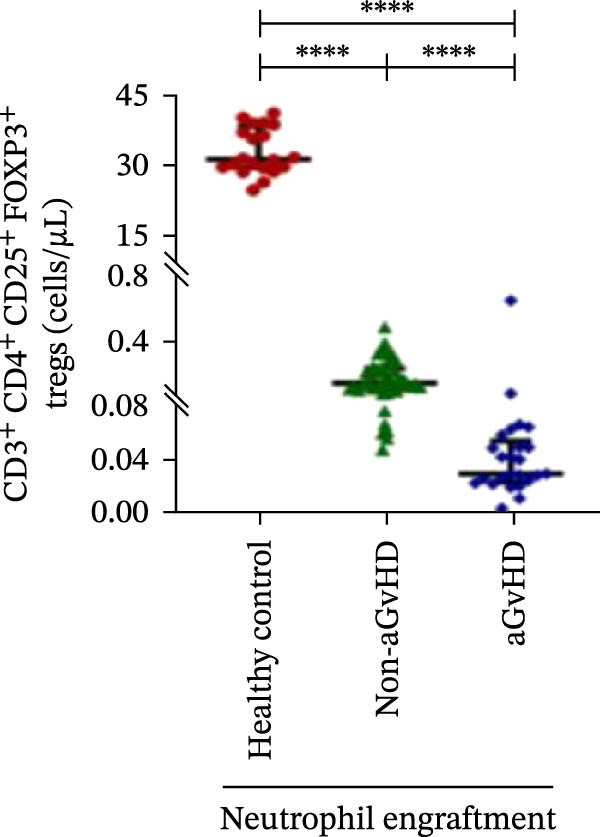
(E)

**Figure:**
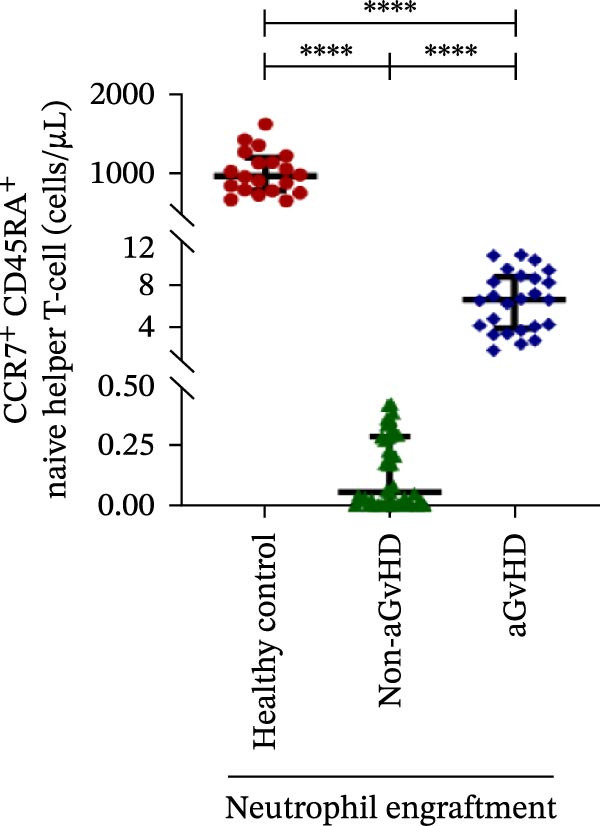
(F)

**Figure:**
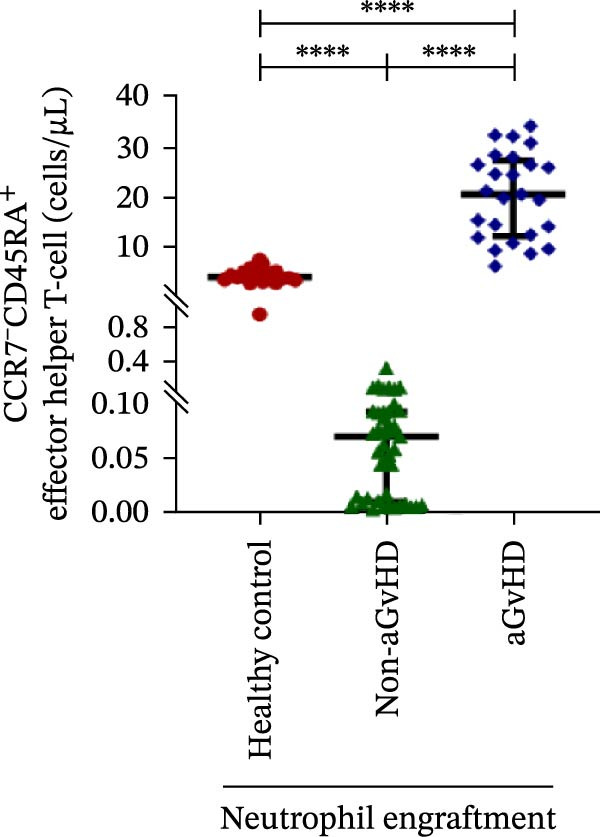
(G)

**Figure:**
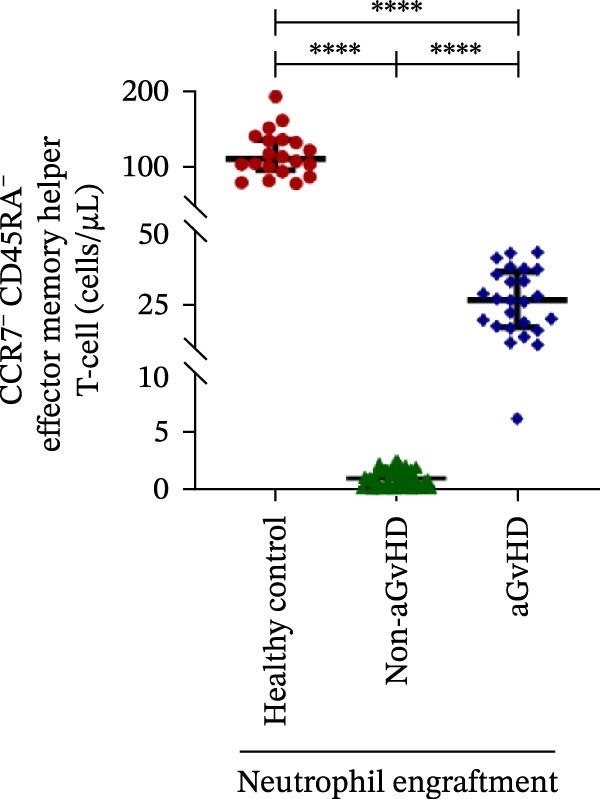
(H)

**Figure:**
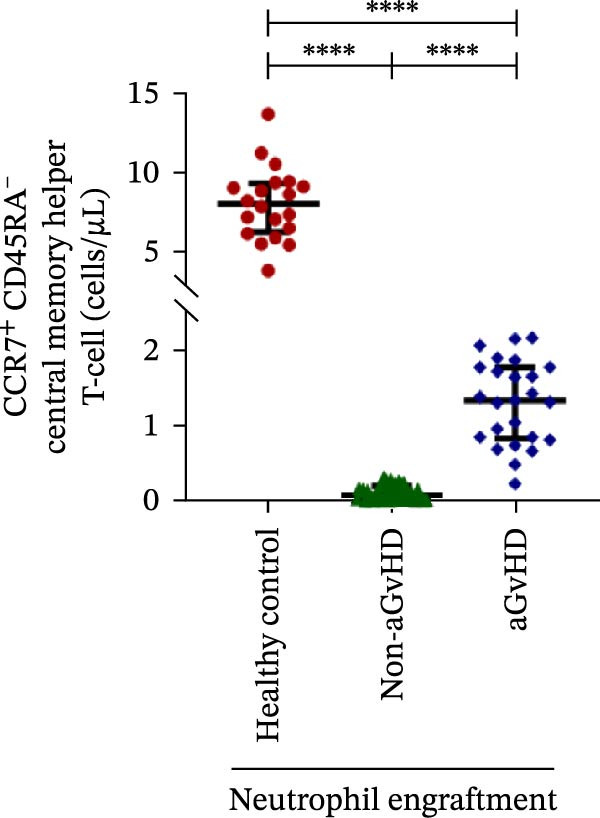
(I)

**Figure:**
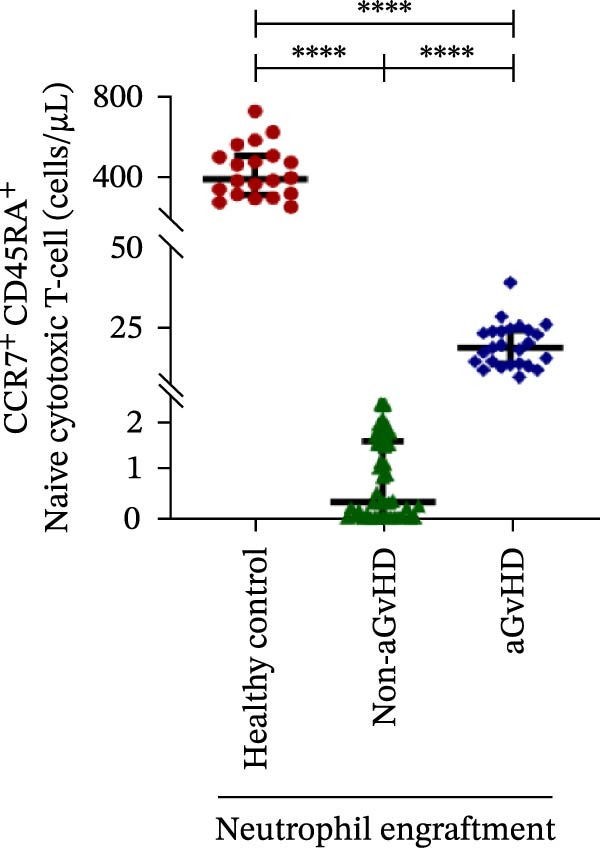
(J)

**Figure:**
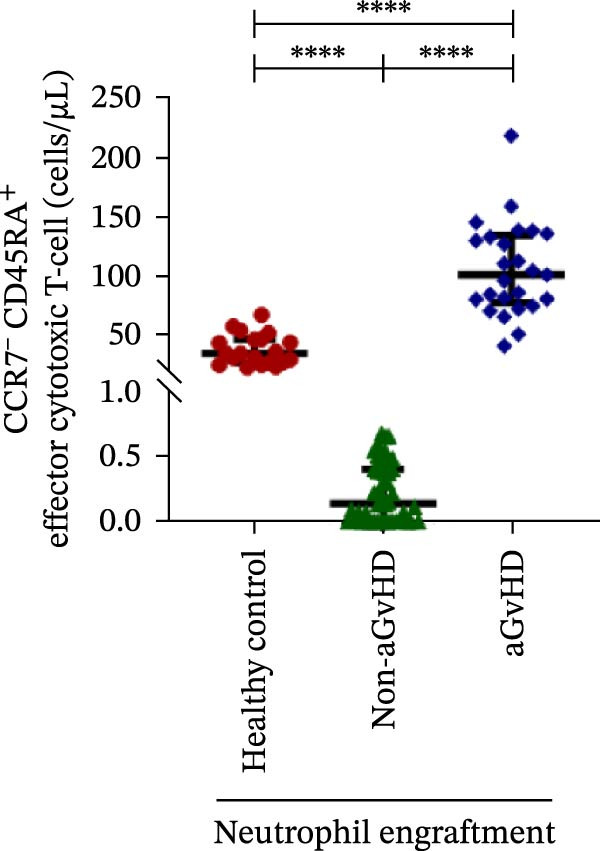
(K)

**Figure:**
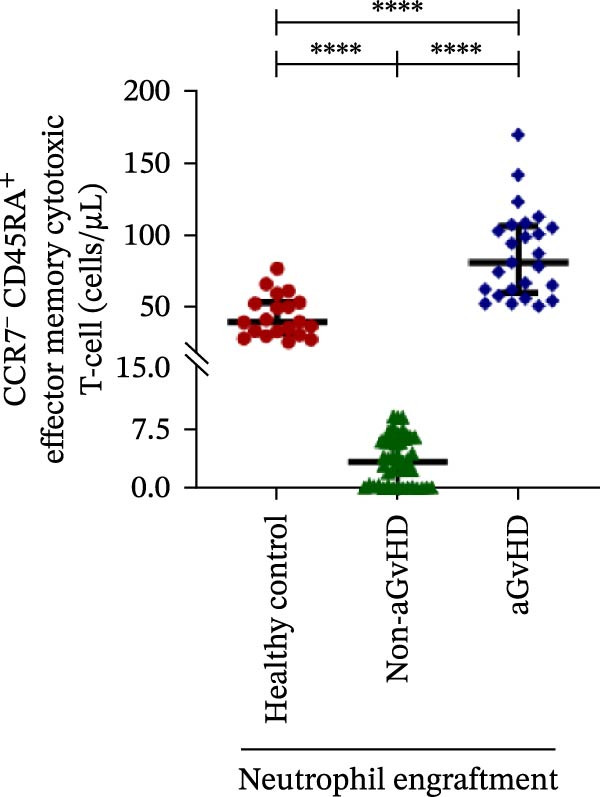
(L)

**Figure:**
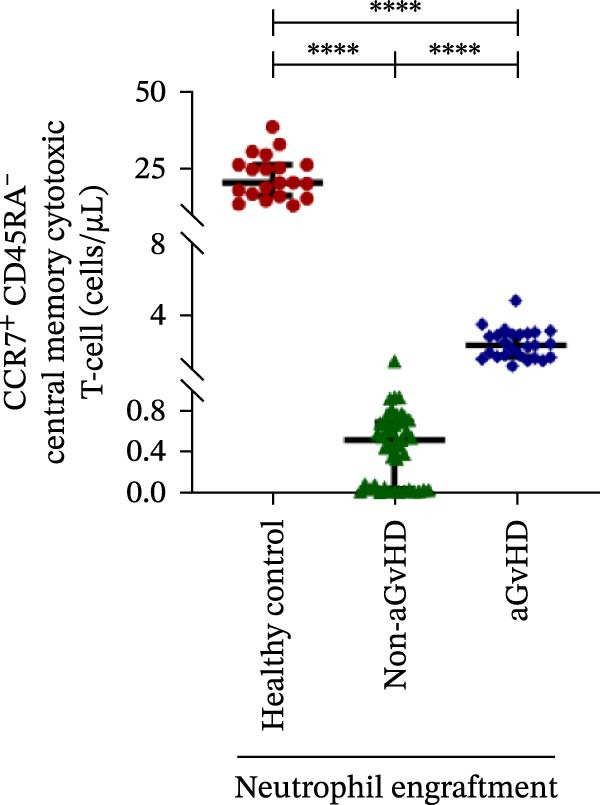
(M)

The Th cell subset analysis showed significantly elevated counts of naïve, effector, effector memory, and central memory cells in aGvHD vs. the non‐aGvHD group (Figure 2F–I). Effector Th cells were also higher than in healthy controls (20.77 vs. 4.28 cells/μL; *p* ≤ 0.0001), indicating selective expansion. Similarly, Tc subsets, particularly effector (101.57 vs. 0.14 cells/μL) and effector memory cells (81.80 vs. 3.38 cells/μL), were markedly increased in aGvHD patients and exceeded levels vs. healthy controls (Figure 2J–M).

Additionally, aGvHD patients had higher levels of proinflammatory Th subsets, including Th1 (14.88 vs. 2.84 cells/μL), Th17 (6.88 vs. 0.0003 cells/μL), and Th22 (1.02 vs. 0.13 cells/μL) (*p* ≤ 0.0001), alongside reduced Th2 (0.14 vs. 1.16 cells/μL) and Th9 (0.09 vs. 0.15 cells/μL) cells (*p* ≤ 0.05). Notably, Th17 cells were also significantly elevated in aGvHD patients compared to healthy controls (6.88 vs. 4.78 cells/μL; *p* ≤ 0.05), suggesting their key role in aGvHD (Figure 3A–E).

Figure 3Profile of subtypes of effector memory Th at the neutrophil engraftment of the patients who either did or did not develop aGvHD in the later phase and healthy control using flow cytometry. The scatter plot represents the absolute count (cells/μL) of (A) Th1, (B) Th2, (C) Th9, (D) Th17, and (E) Th22. Data are presented as the median with interquartile range for 20 healthy control and 70 patients (aGvHD = 25; non‐aGvHD = 45). Statistical analysis: Mann–Whitney test;  ^∗^ ≤ 0.05;  ^∗∗∗∗^ ≤ 0.0001. aGvHD, acute graft‐versus‐host‐disease; Th, helper T‐cell.

**Figure:**
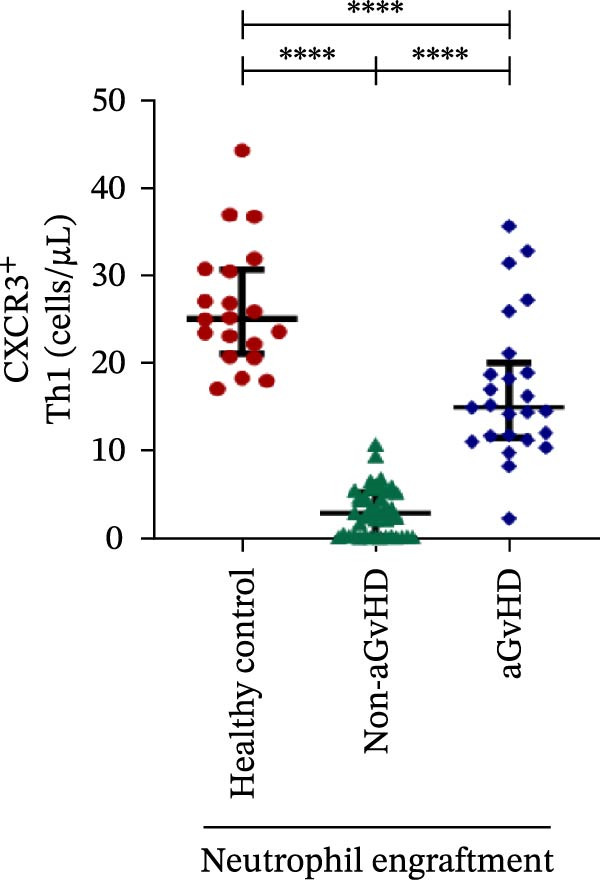
(A)

**Figure:**
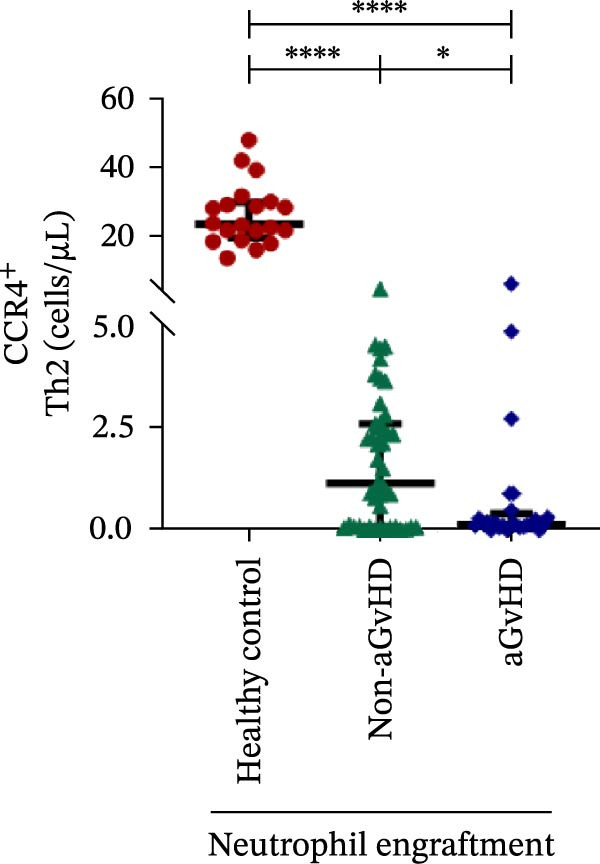
(B)

**Figure:**
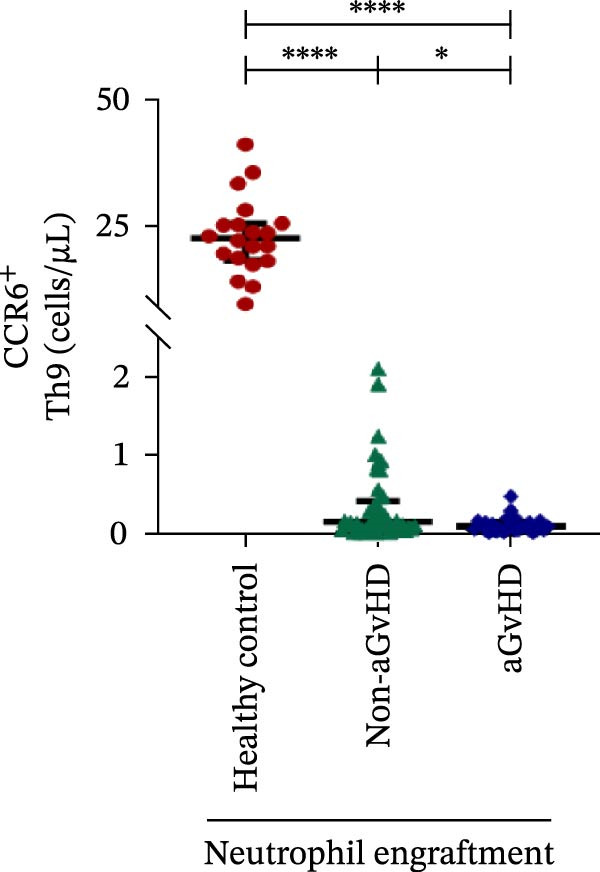
(C)

**Figure:**
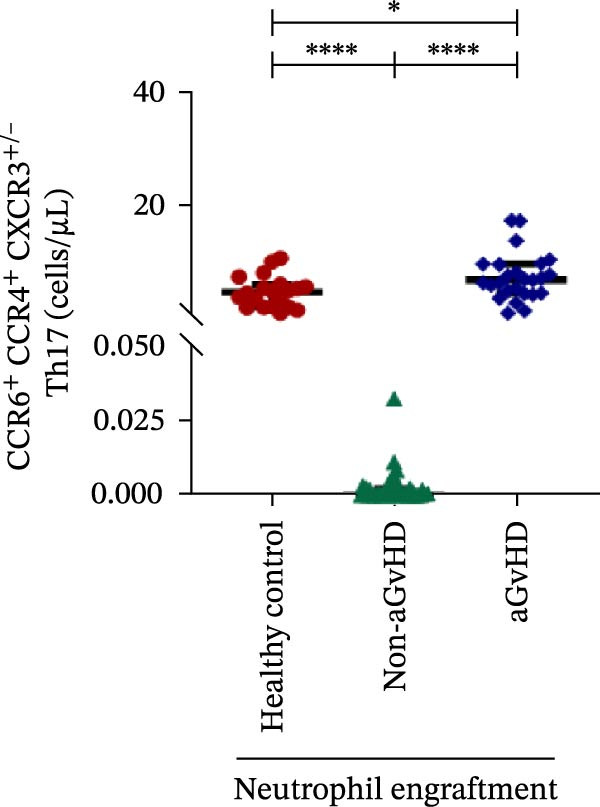
(D)

**Figure:**
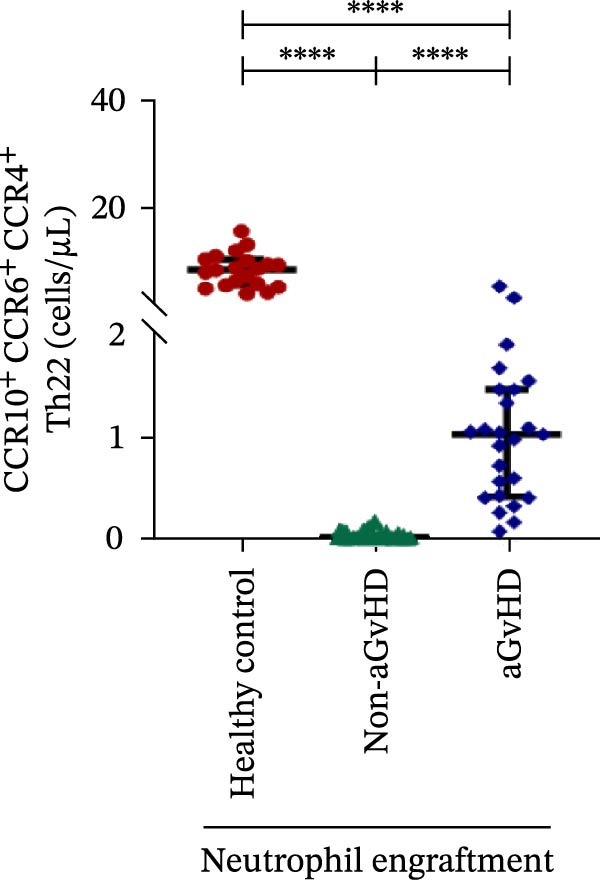
(E)

### 3.3. NK Cell Dynamics at the Neutrophil Engraftment Associated With aGvHD Onset

We analyzed NK cells and their subsets in transplant recipients at the time of neutrophil engraftment to assess their potential in predicting aGvHD onset. The gating strategy used for NK cell enumeration and subtyping is depicted in Figure S2A.

A comparative analysis of neutrophil engraftment profiles between patients who later developed aGvHD and those who did not revealed a significantly higher NK cell count in the aGvHD cohort (436.73 cells/μL vs. 138.86 cells/μL; *p* ≤ 0.0001) (Figure 4A). This increase was primarily attributed to a significant expansion of cytotoxic NK cells (387.87 cells/μL vs. 113.78 cells/μL; *p* ≤ 0.0001), accompanied by a concurrent reduction in regulatory NK cells (2.59 cells/μL vs. 12.96 cells/μL; *p* ≤ 0.0001) in aGvHD patients compared to the non‐aGvHD cohort (Figure 4B–D). Interestingly, NK cell counts were also significantly elevated in the aGvHD cohort compared to healthy controls (436.74 cells/μL vs. 255.18 cells/μL; *p* ≤ 0.0001), with a similar trend observed for cytotoxic NK cells (387.87 cells/μL vs. 174.73 cells/μL; *p* ≤ 0.0001) (Figure 4A,C).

Figure 4Natural killer cell, dendritic cell, and their subtypes at the neutrophil engraftment of the patients who either did or did not develop aGvHD in the later phase and healthy control using flow cytometry. The scatter plot represents the absolute count (cells/μL) of (A) CD3− CD56+ NK cells, (B) CD56bright CD16− regulatory NK cells, (C) CD56dim CD16+ cytotoxic NK cells, (D) CD56bright CD16− KIR− regulatory NK cells, (E) CD56dim CD16+ KIR+ regulatory NK cells, (F) KIR−/KIR+ regulatory NK cells, (G) CD56dim CD16+ KIR− cytotoxic NK cells, (H) CD56dim CD16+ KIR+ cytotoxic NK cells, (I) KIR−/KIR+ cytotoxic NK cells, (J) CD11c+ HLA‐DR+ dendritic cells, (K) CD11c+ CD123+ pDC, (L) CD11c+ CD123− cDC, (M) CD141+ CD1c− cDC1, and (N) CD141− CD1c+ cDC2. Data are presented as the median with interquartile range for 20 healthy control and 70 patients (aGvHD = 25; non‐aGvHD = 45). Statistical analysis: Mann–Whitney test;  ^∗^ ≤ 0.05;  ^∗∗^ ≤ 0.01;  ^∗∗∗^ ≤ 0.001;  ^∗∗∗∗^ ≤ 0.0001. aGvHD, acute graft‐versus‐host‐disease; cDC, conventional dendritic cell; KIR, killer immunoglobulin‐like receptors; NK, natural killer cell; pDC, plasmacytoid dendritic cell.

**Figure:**
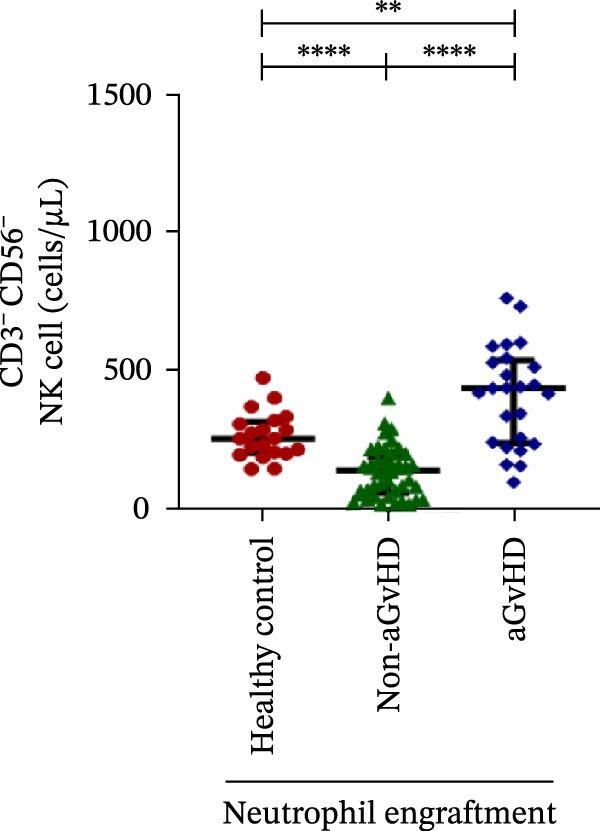
(A)

**Figure:**
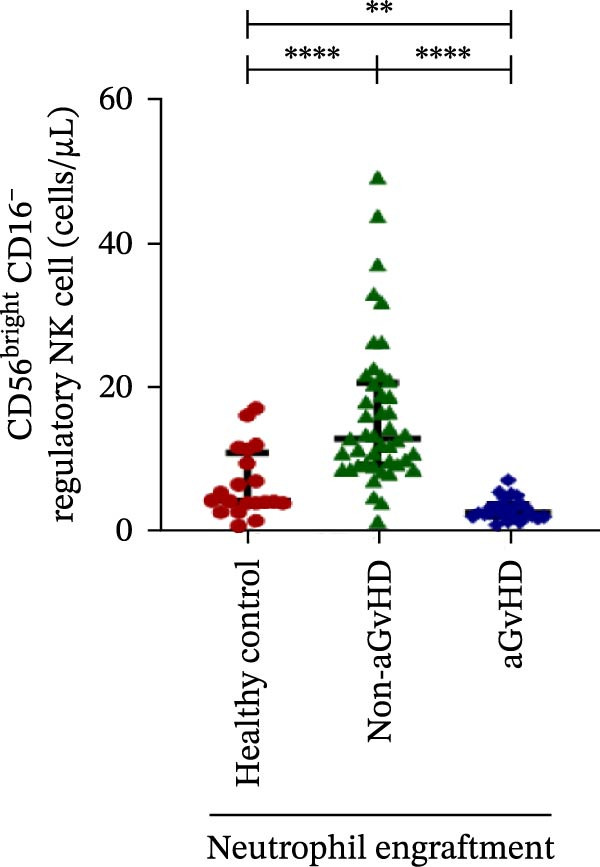
(B)

**Figure:**
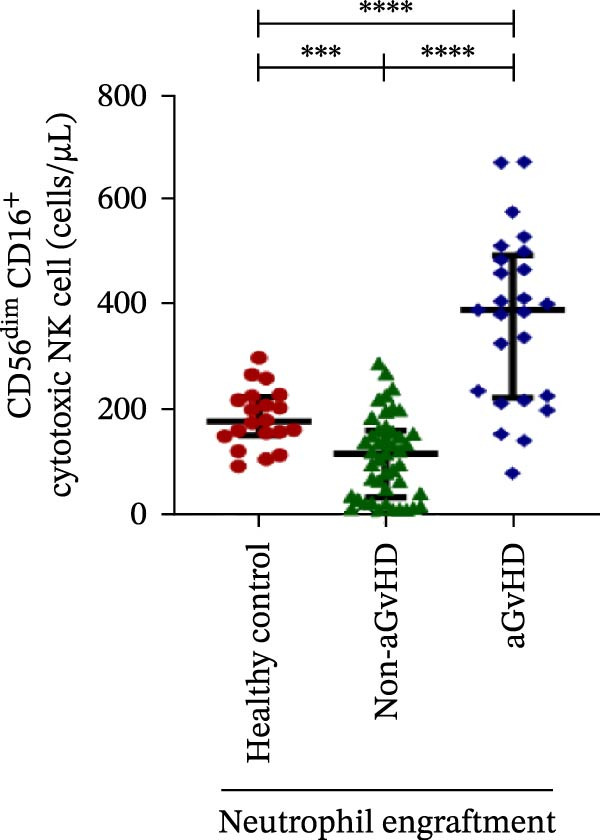
(C)

**Figure:**
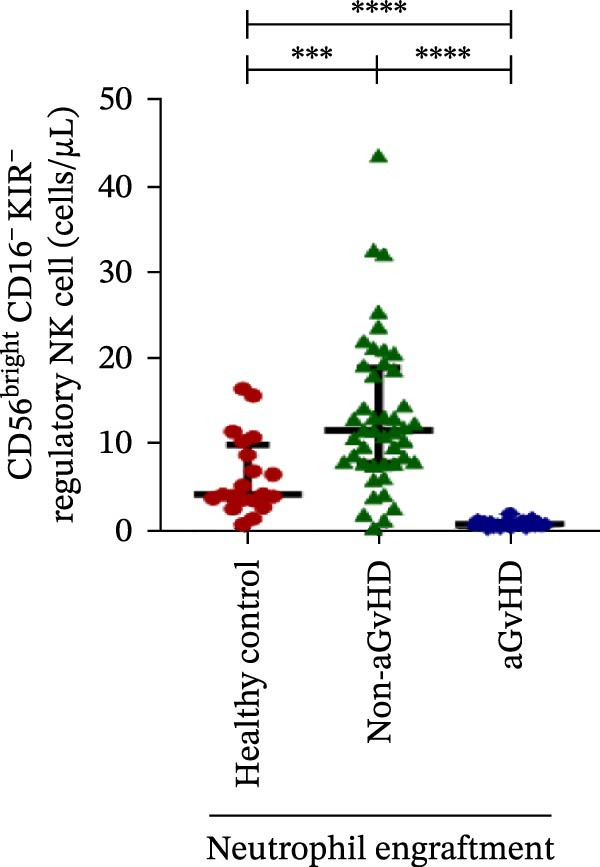
(D)

**Figure:**
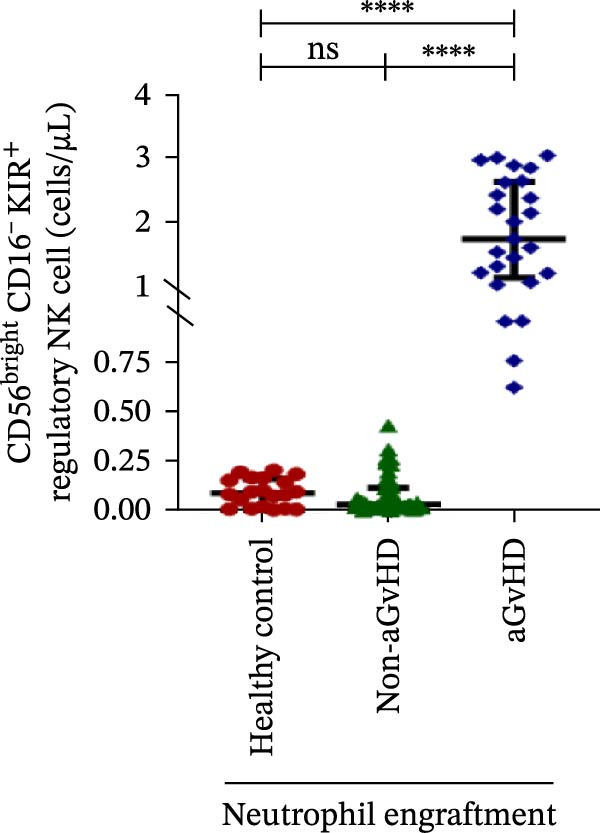
(E)

**Figure:**
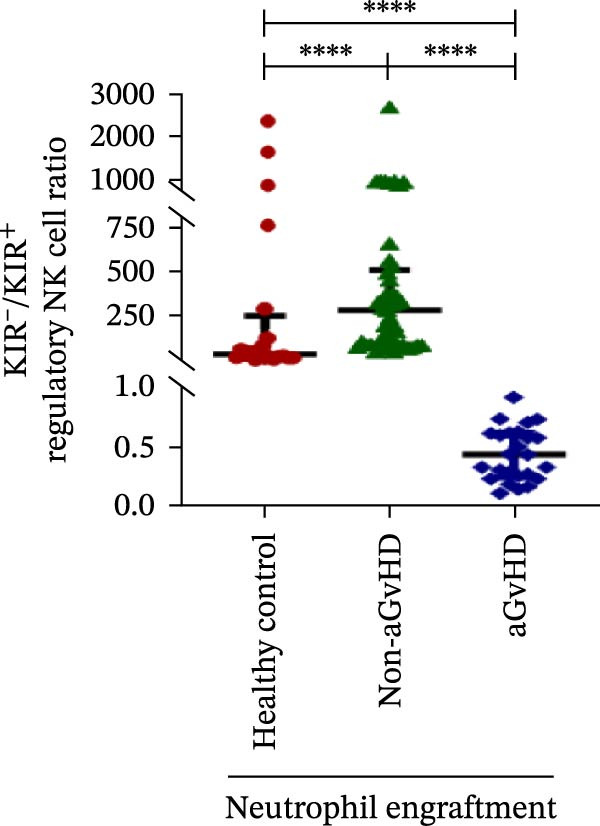
(F)

**Figure:**
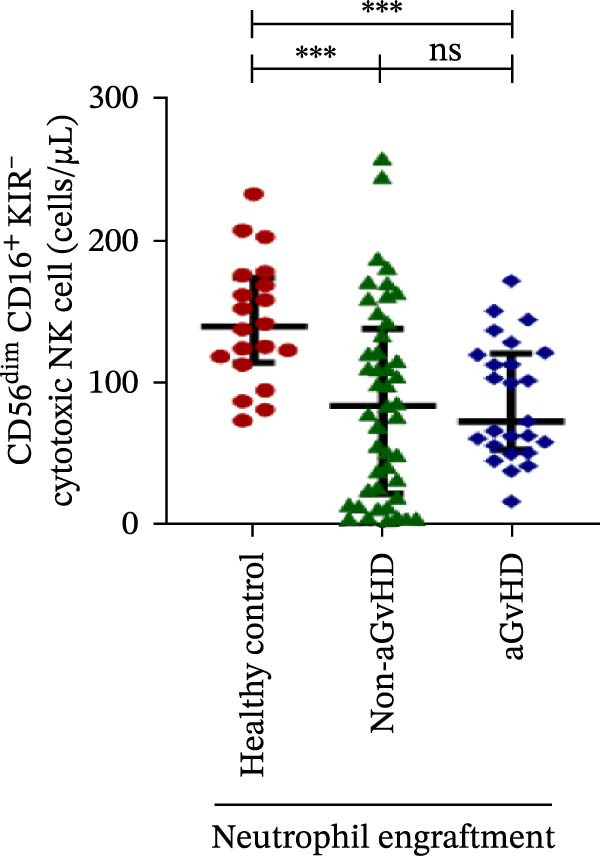
(G)

**Figure:**
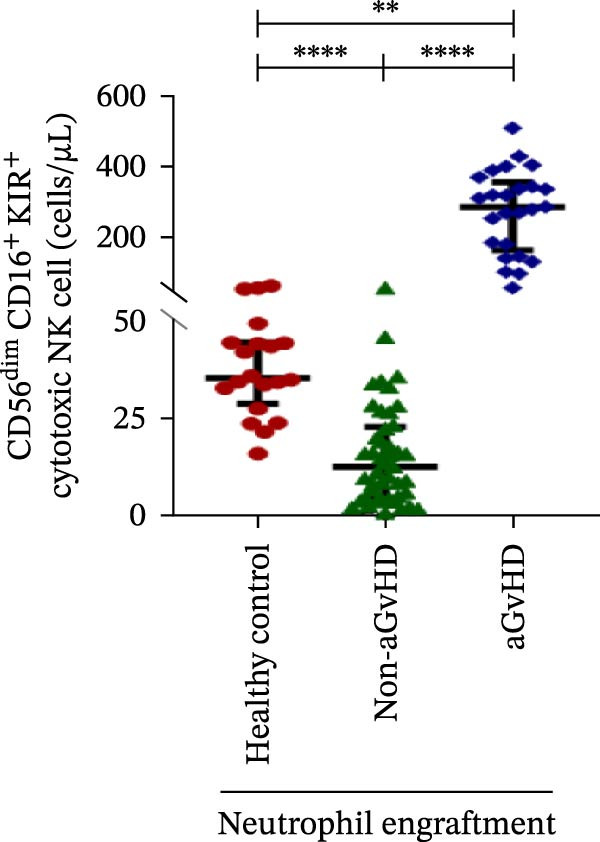
(H)

**Figure:**
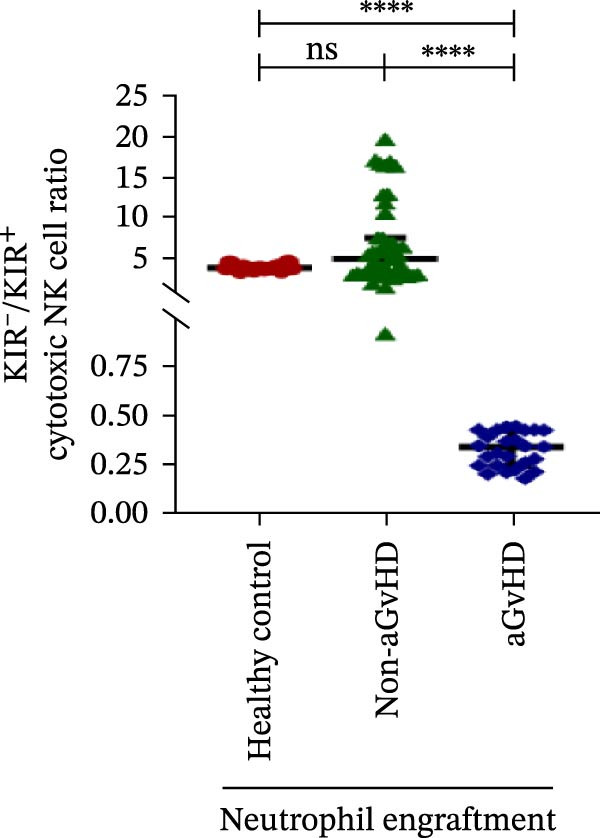
(I)

**Figure:**
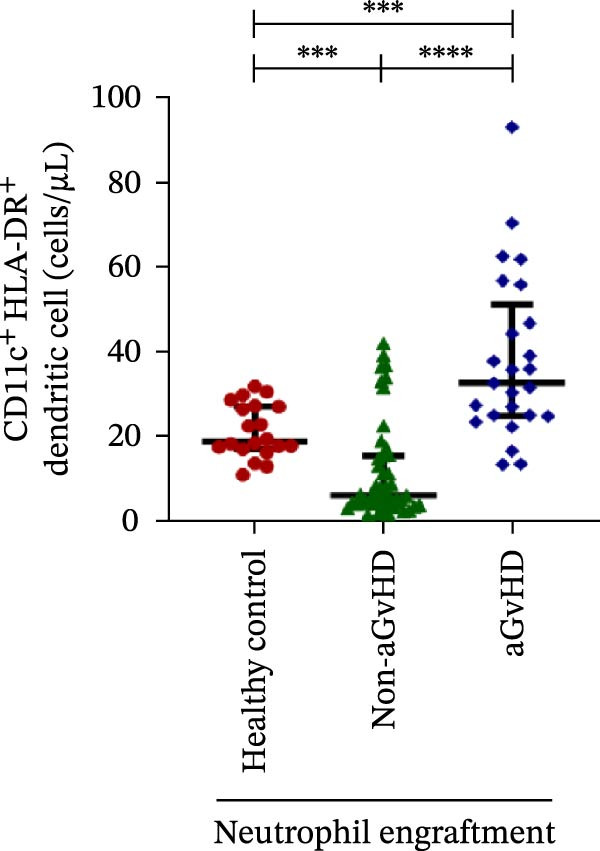
(J)

**Figure:**
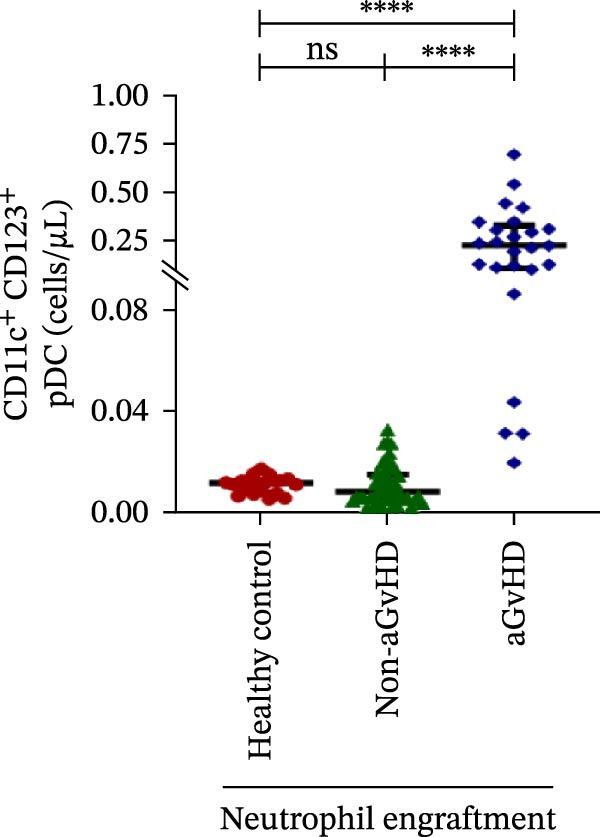
(K)

**Figure:**
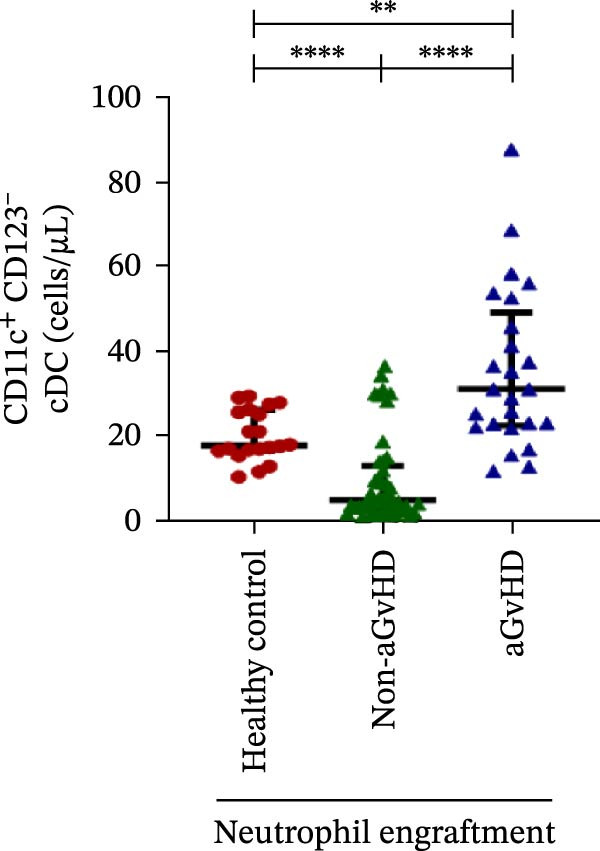
(L)

**Figure:**
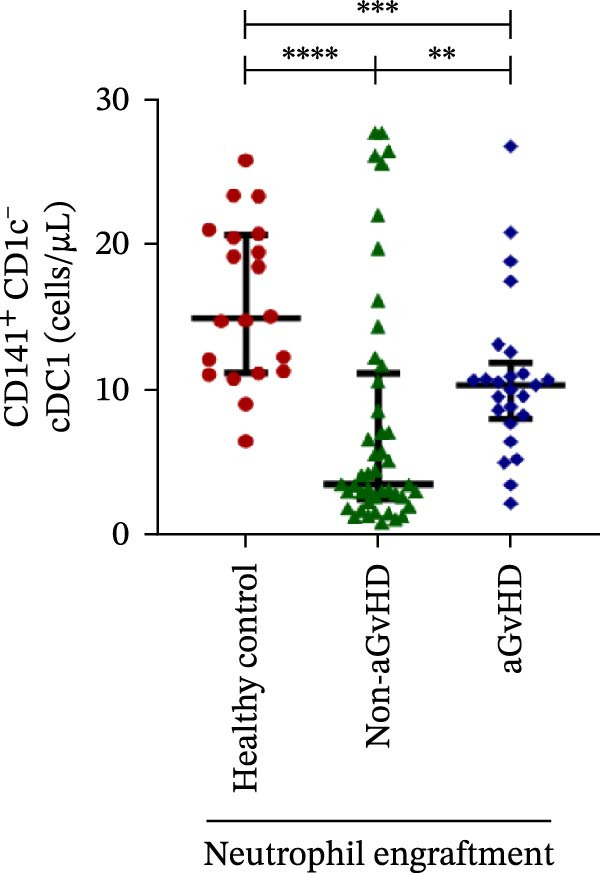
(M)

**Figure:**
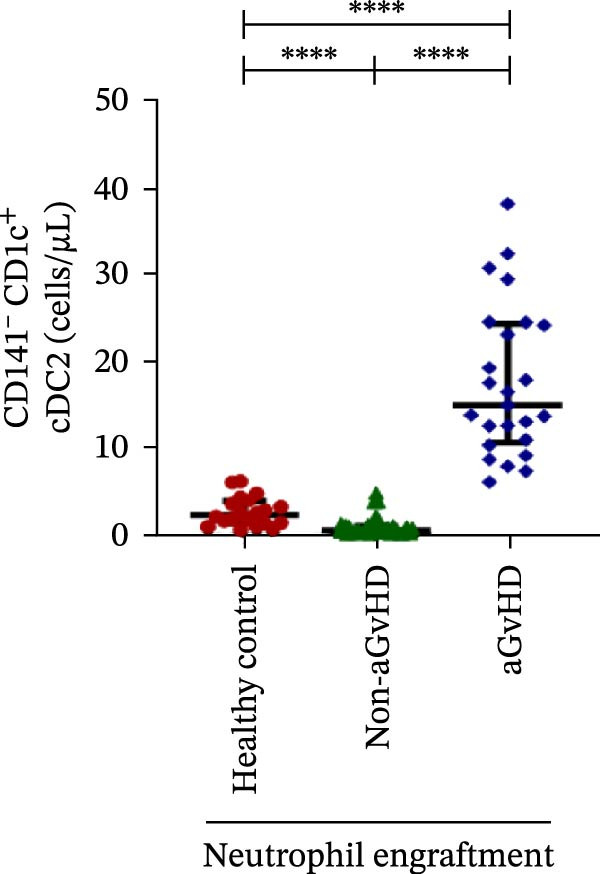
(N)

Moreover, at the time of neutrophil engraftment, the aGvHD cohort exhibited a significantly higher fraction of regulatory NK cells expressing KIR (1.76 cells/μL vs. 0.03 cells/μL; *p* ≤ 0.0001), while the fraction of regulatory NK cells lacking KIR expression was markedly reduced (0.73 cells/μL vs. 11.55 cells/μL; *p* ≤ 0.0001) compared to the non‐aGvHD cohort (Figure 4D, E). This was reflected in a significant decrease in the KIR^−^/KIR^+^ regulatory NK cell ratio (0.46 vs. 292.68; *p* ≤ 0.0001) in aGvHD patients (Figure 4F).

Notably, no significant difference was observed in the fraction of KIR^−^ cytotoxic NK cells between the aGvHD and non‐aGvHD cohorts (73.11 cells/μL vs. 84.10 cells/μL; *p* 0.6176). However, the fraction of KIR^+^ cytotoxic NK cells was significantly elevated in aGvHD patients (286.24 cells/μL vs. 12.60 cells/μL; *p* ≤ 0.0001) (Figure 4G,H). Consequently, the KIR^−^/KIR^+^ cytotoxic NK cell ratio was significantly reduced in the aGvHD cohort vs. non‐aGvHD cohort (0.34 vs. 4.93; *p* ≤ 0.0001) (Figure 4I).

### 3.4. Elevated DCs With the Predominance of cDC2 Drive aGvHD Onset

Patients who developed aGvHD later exhibited significantly higher DC counts compared to both the non‐aGvHD cohort (38.27 cells/µL vs. 11.98 cells/µL; *p* ≤ 0.0001) and healthy controls (38.26 cells/µL vs. 21.51 cells/µL; *p* ≤ 0.001) (Figure 4J). Notably, both pDC and cDC were significantly elevated in the aGvHD cohort relative to the non‐aGvHD cohort (pDCs: 0.23 cells/µL vs. 0.008 cells/µL; *p* ≤ 0.0001; cDCs: 31.25 cells/µL vs. 5.05 cells/µL; *p* ≤ 0.0001) as well as healthy controls (pDCs: 0.23 cells/µL vs. 0.012 cells/µL; *p* ≤ 0.0001; cDCs: 31.25 cells/µL vs. 17.98 cells/µL; *p* ≤ 0.0001) (Figure 4K,L).

Within the cDC subset, both cDC1 and cDC2 fractions were elevated in aGvHD compared to the non‐aGvHD cohort (cDC1:10.31 cells/µL vs. 3.46 cells/µL; *p* ≤ 0.01; cDC2:14.98 cells/µL vs. 0.55 cells/µL; *p* ≤ 0.0001). Furthermore, cDC2 was significantly more abundant in the aGvHD cohort than in healthy controls (14.98 cells/µL vs. 2.35 cells/µL; *p* ≤ 0.0001) (Figure 4M,N).

### 3.5. Elevated B Cell Count at the Neutrophil Engraftment Predicted aGVHD Onset in the Transplant Recipients

The gating strategy for B cell enumeration and subtyping is depicted in Figure S3. The absolute B cell counts were significantly elevated in patients who later developed aGvHD compared to non‐aGvHD recipients (18.68 vs. 0.52 cells/µL; *p* ≤ 0.0001) (Figure S5B). This increase extended across all B cell subtypes, including naïve (9.39 vs. 0.31 cells/µL), unswitched memory (USM) (0.25 vs. 0.02 cells/µL), switched memory (SM) (0.43 vs. 0.037 cells/µL), and double negative SM (DNSM) B cell (2.51 vs. 0.069 cells/µL), all showing significant differences (*p* ≤ 0.0001), suggesting broad B cell expansion in the aGvHD cohort (Figure S4A–E).

### 3.6. Imbalance in Cytokines at the Neutrophil Engraftment Predicted aGvHD Onset in the Transplant Recipients

Our analysis showed that patients who went on to develop aGvHD had a distinct proinflammatory cytokine profile at engraftment, with significantly elevated levels of IFN‐γ, IL‐1β, IP‐10, TNF‐α, IL‐17A, IL‐12 (p70), IL‐6, IL‐5, RANTES, MIP‐1α, and MIP‐1β, alongside reduced levels of anti‐inflammatory cytokines such as IL‐2, IL‐4, and IL‐10 (Figure 5A–N). This imbalance, which is marked by heightened inflammatory signaling and diminished regulatory cytokines, distinguished aGvHD patients from both healthy controls and non‐aGvHD transplant recipients.

Figure 5Cytokines and chemokines profile at the neutrophil engraftment of the patients who either did or did not develop aGvHD in the later phase and healthy control. The scatter plot represents the concentration (pg/ml) of (A) IFN‐γ, (B) IL‐1β, (C) IP‐10 (ng/mL), (D) TNF‐α, (E) IL‐17α, (F) IL‐12 (p70), (G) IL‐6, (H) IL‐2, (I) IL‐4, (J) IL‐5, (K) IL‐10, (L) RANTES, (M) MIP‐1α, and (N) MIP‐1β. Data are presented as the median with interquartile range for 20 healthy control and 70 patients (aGvHD = 25; non‐aGvHD = 45). Statistical analysis: Mann–Whitney test;  ^∗∗∗∗^ ≤ 0.0001. aGvHD, acute graft‐versus‐host‐disease; IFN‐γ, interferon‐ γ; IL, interleukin; MIP, macrophage inflammatory protein; RANTES, regulated on activation, normal T‐cell expressed and secreted.

**Figure:**
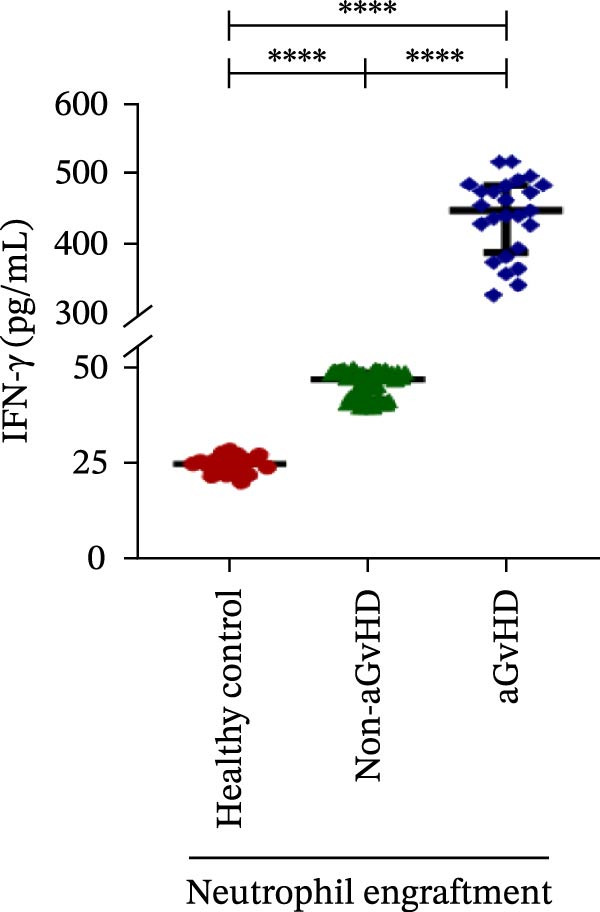
(A)

**Figure:**
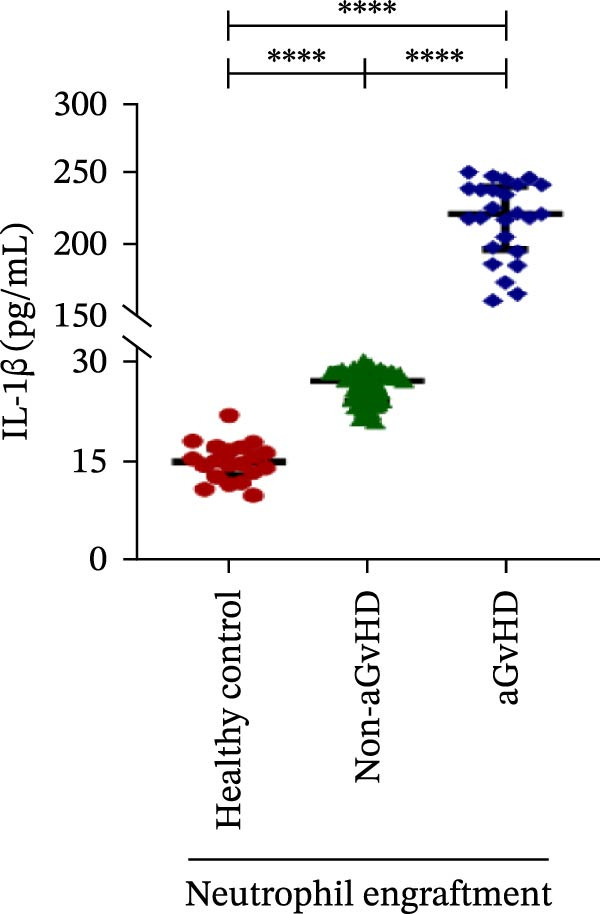
(B)

**Figure:**
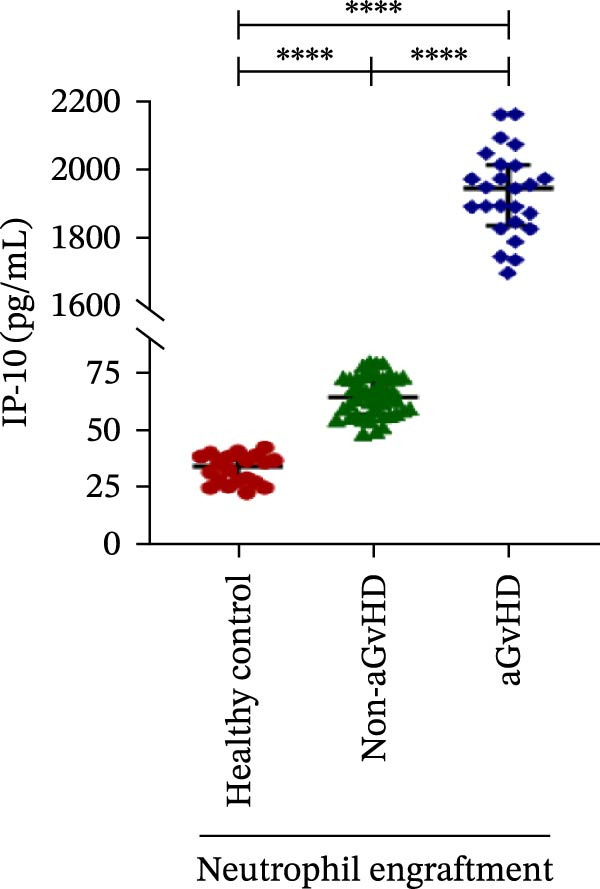
(C)

**Figure:**
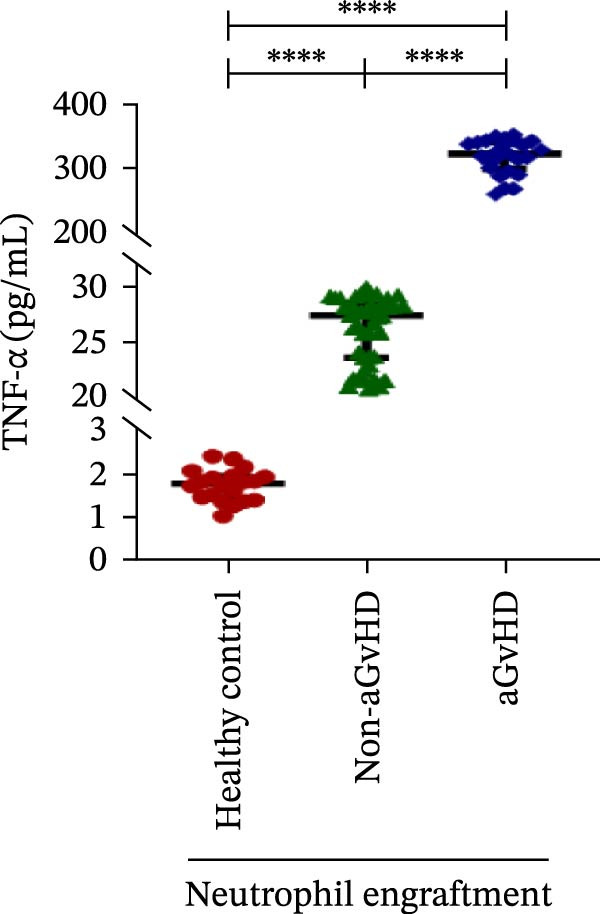
(D)

**Figure:**
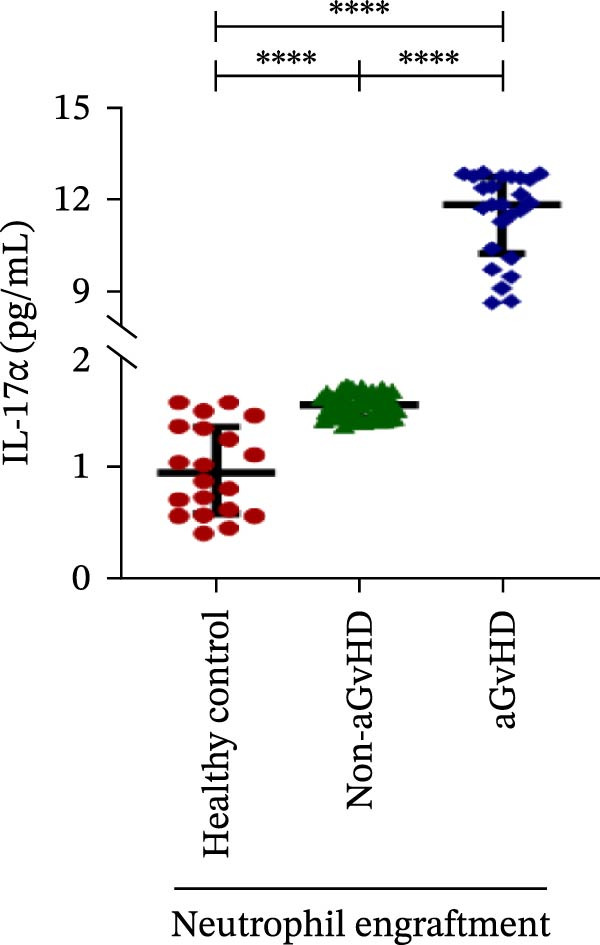
(E)

**Figure:**
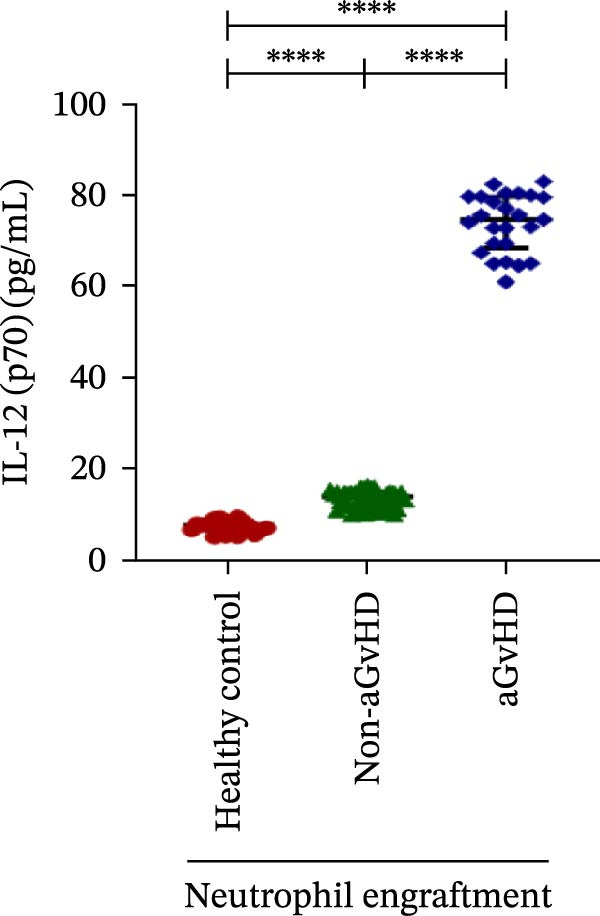
(F)

**Figure:**
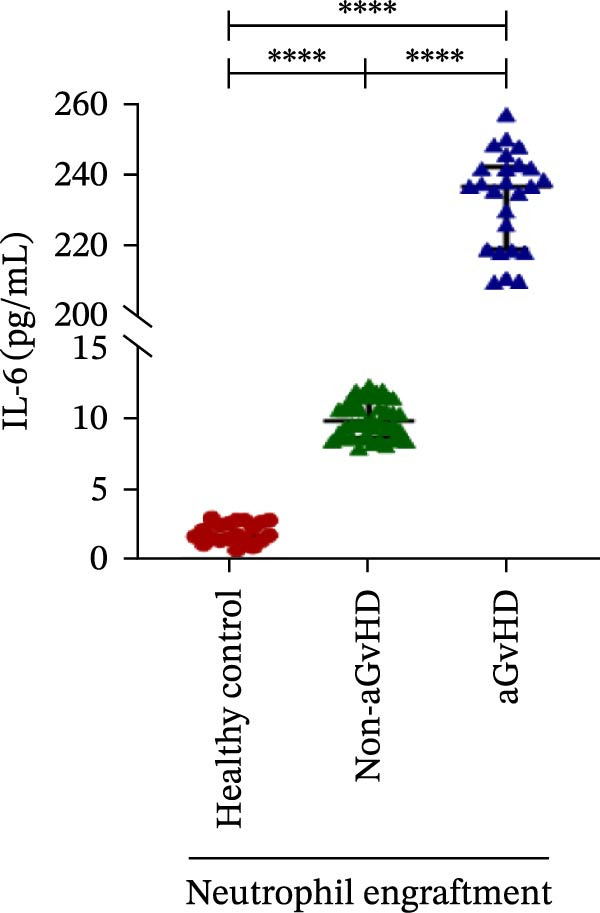
(G)

**Figure:**
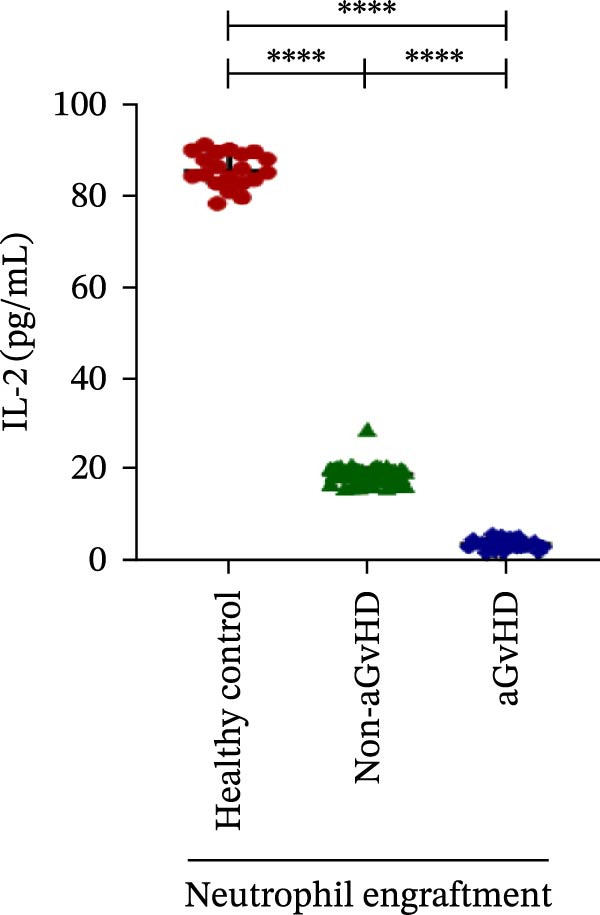
(H)

**Figure:**
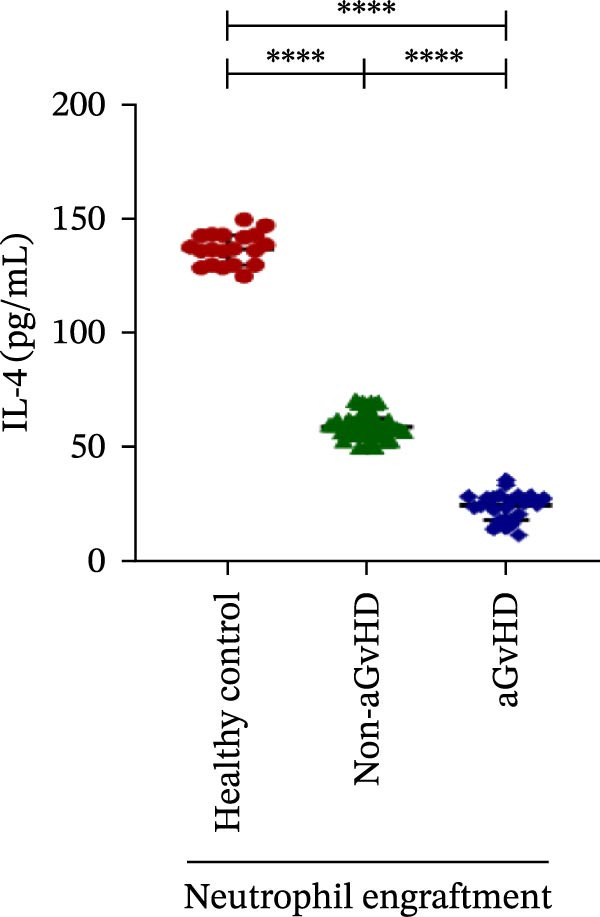
(I)

**Figure:**
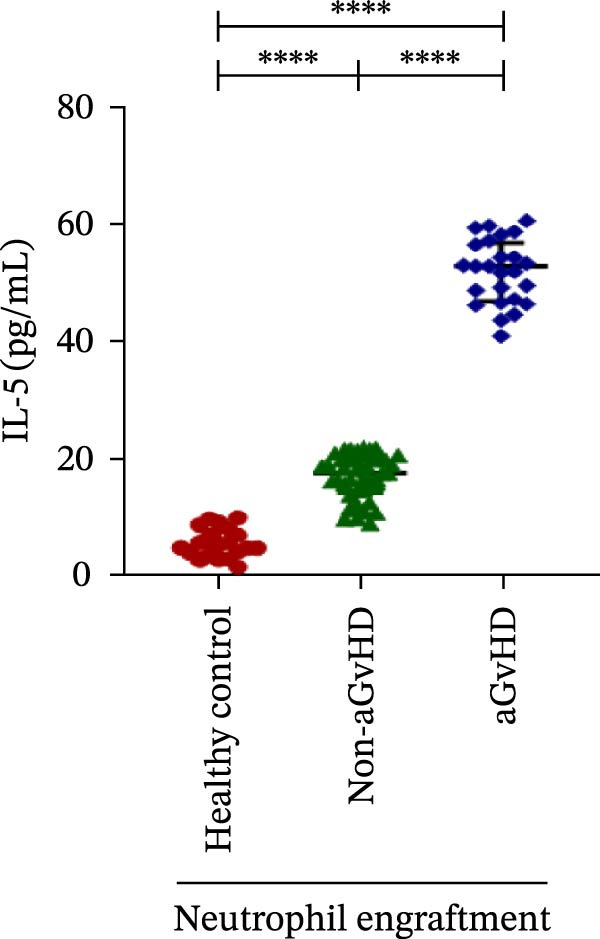
(J)

**Figure:**
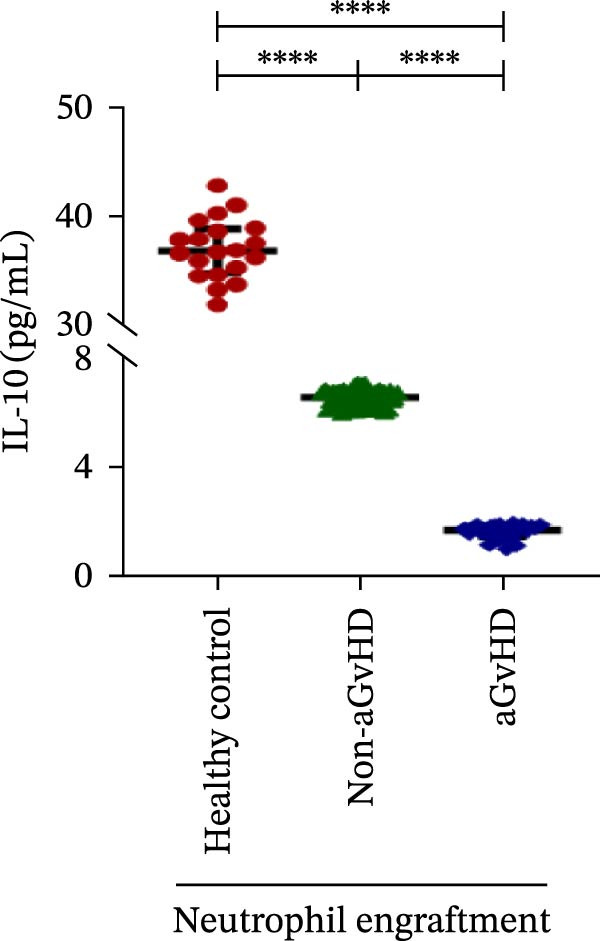
(K)

**Figure:**
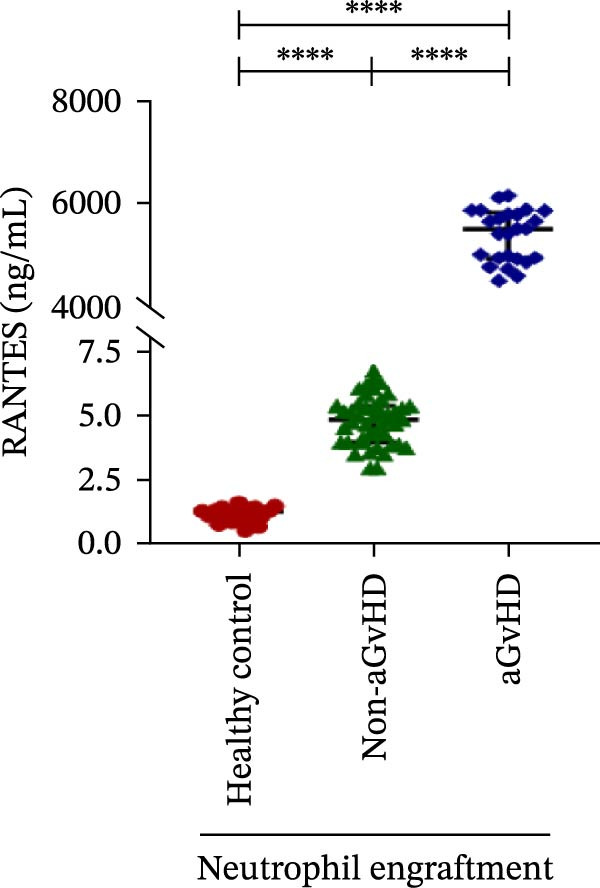
(L)

**Figure:**
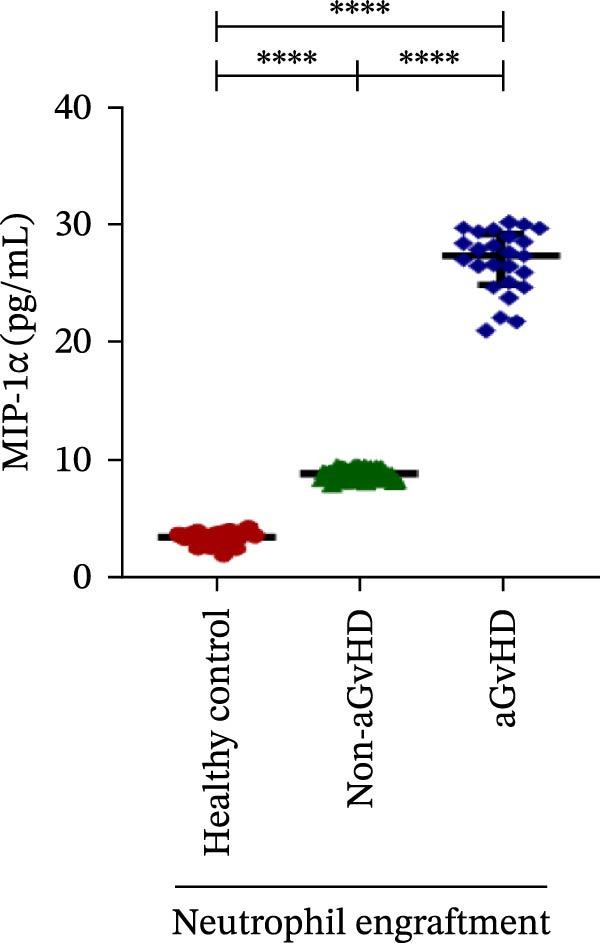
(M)

**Figure:**
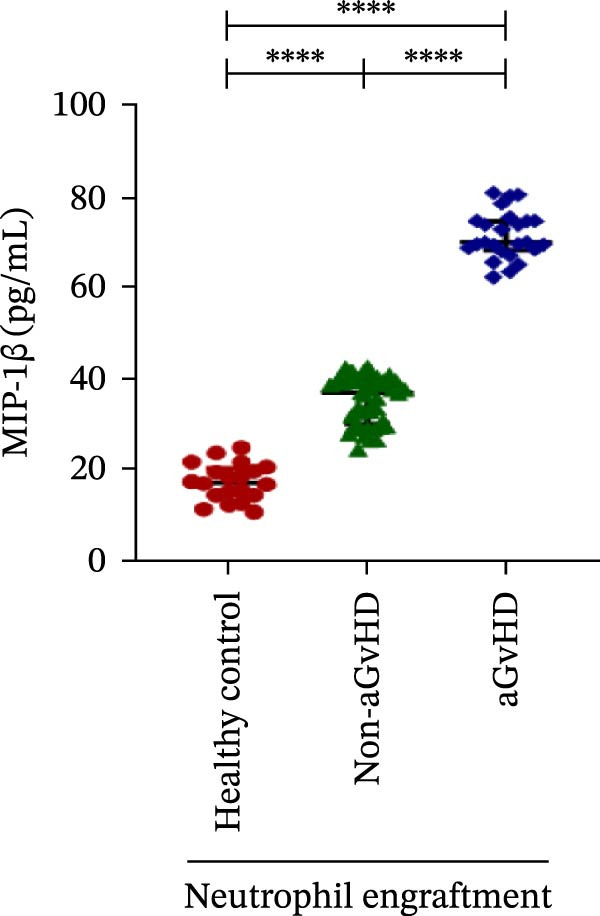
(N)

### 3.7. ML Algorithm Identified Cytokine Levels as Better Predictive Markers Than Cellular Parameters at the Time of Neutrophil Engraftment

Our ML analysis of 48 immune parameters, comprising 34 immune cell subsets and 14 cytokines, was at the time of neutrophil engraftment. Given the complexity and high dimensionality of the dataset, ML was employed to integrate and model these features, supporting the development of predictive tools for early risk stratification and personalized posttransplant care (Figure 6A).

Figure 6Integration of the ML algorithm in developing a predictive model for aGvHD using the cellular and cytokine profiles of transplant recipients at neutrophil engraftment. (A) Schematic representation of the methodology adopted for the ML approach. The correlation map shows (B) a combination of cytokines and cellular profiles. (C) Cytokine profile only. (D) Cellular profile only. (E) T‐cell and their subtypes. (F) NK cells and their subtypes. (G) Dendritic cells and their subtypes. (H) B‐cell and their subtypes. The UMAP shows (I) cytokines. (J) Immune cells. (K) T‐cell. (L) NK cells. (M) Dendritic cells. (N) B‐cell of transplant recipients at neutrophil engraftment for the identification of predictive marker for aGvHD. NK, natural killer.

**Figure:**
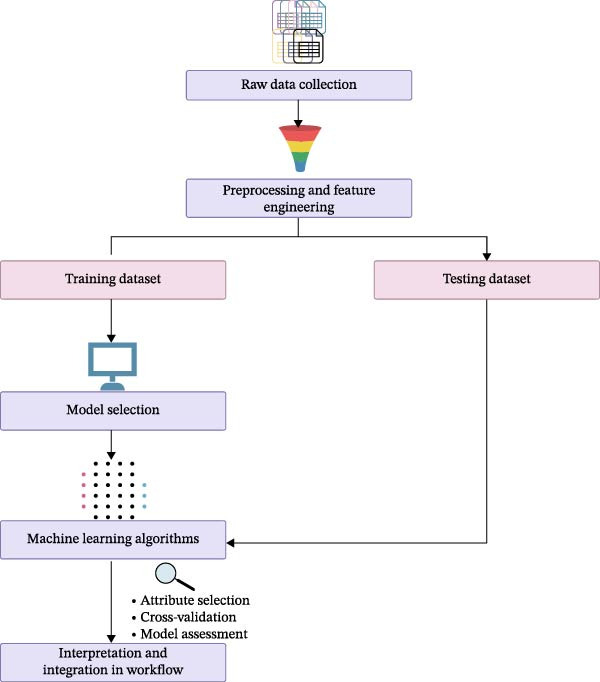
(A)

**Figure:**
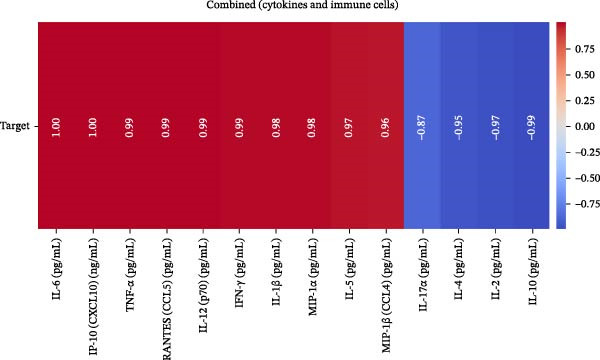
(B)

**Figure:**
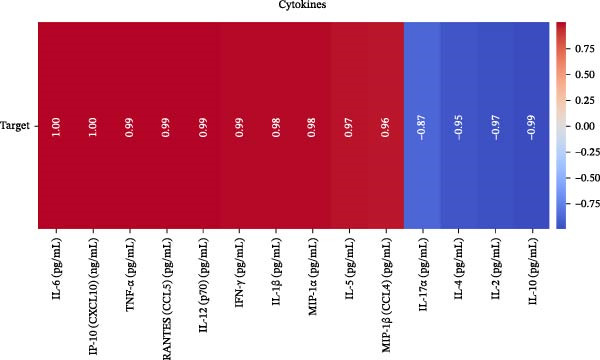
(C)

**Figure:**
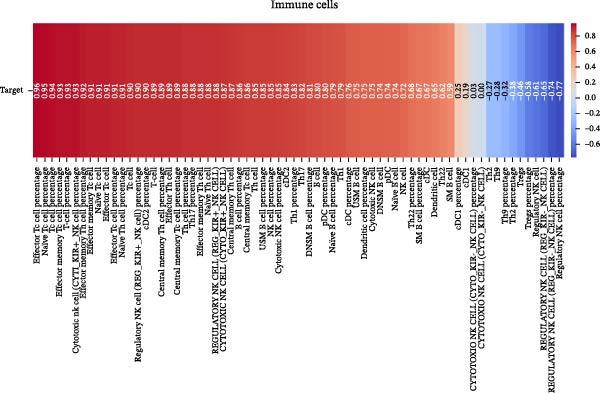
(D)

**Figure:**
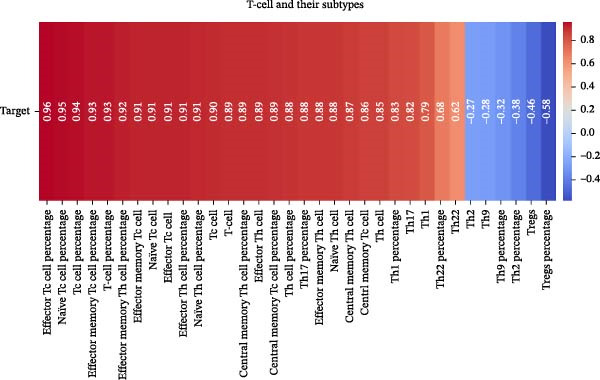
(E)

**Figure:**
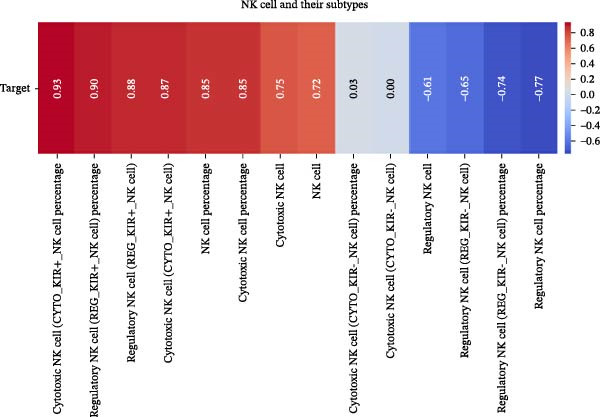
(F)

**Figure:**
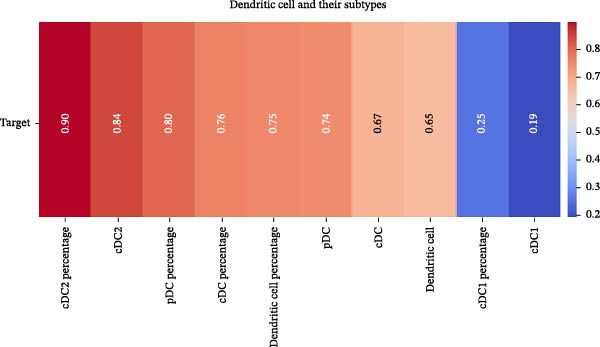
(G)

**Figure:**
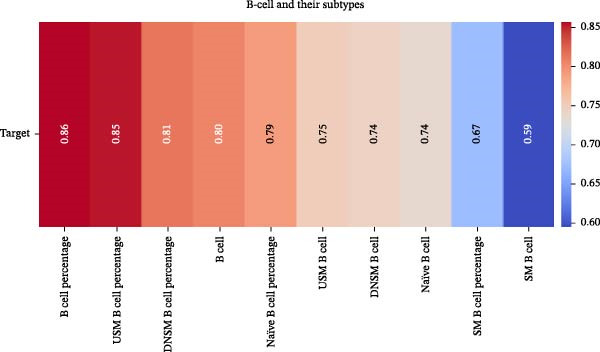
(H)

**Figure:**
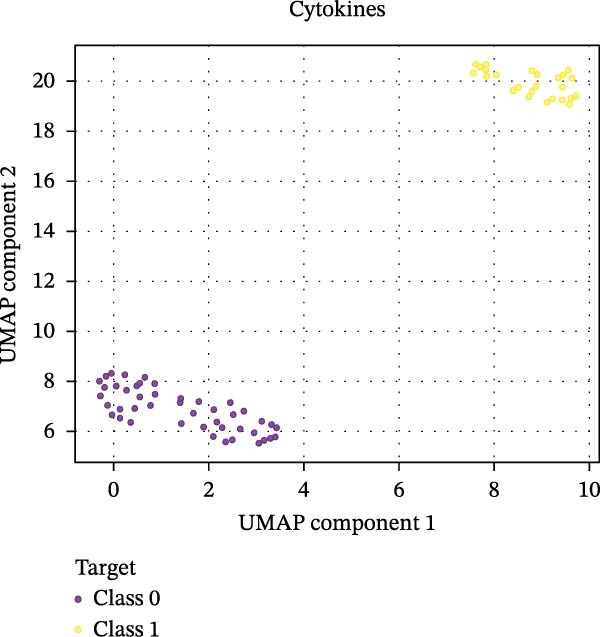
(I)

**Figure:**
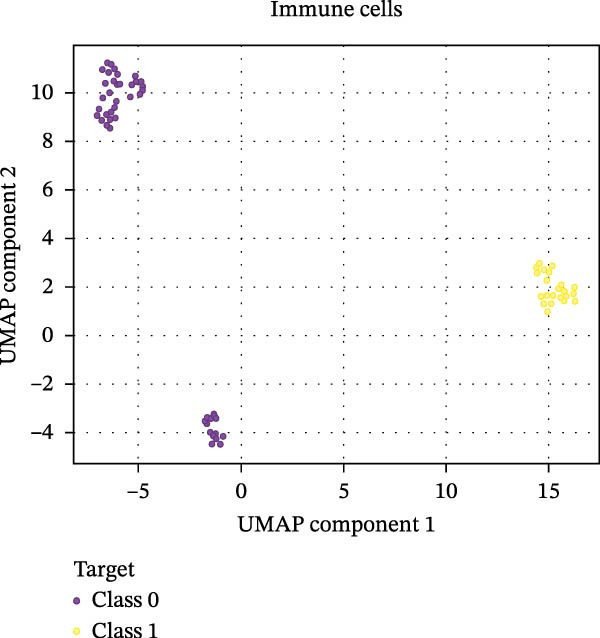
(J)

**Figure:**
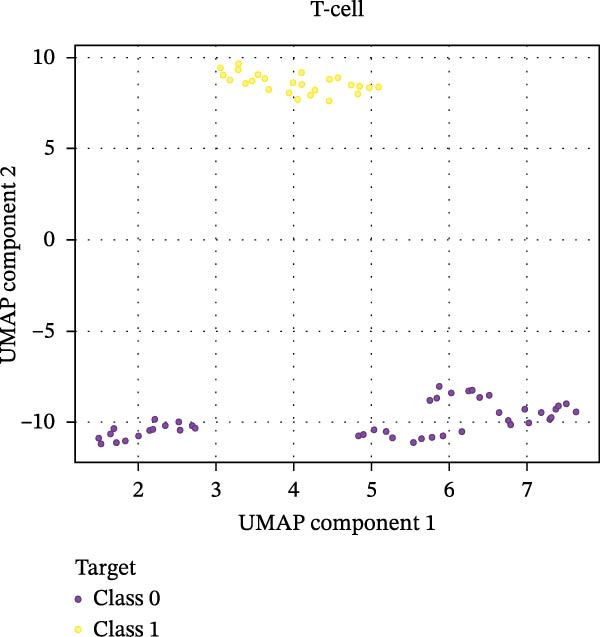
(K)

**Figure:**
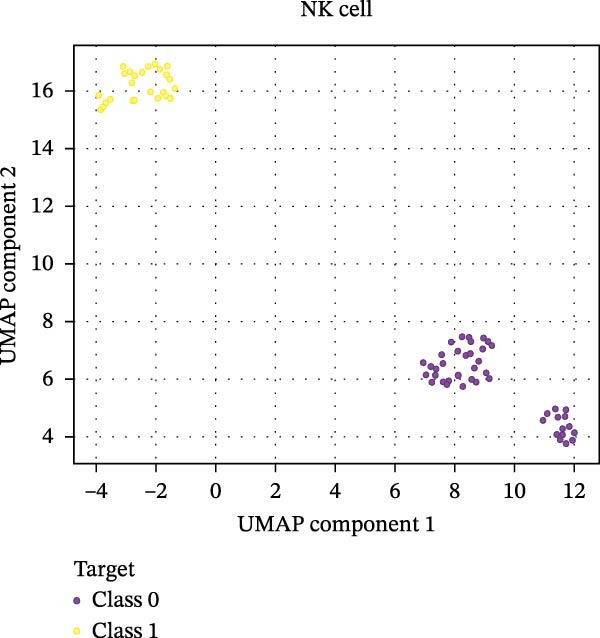
(L)

**Figure:**
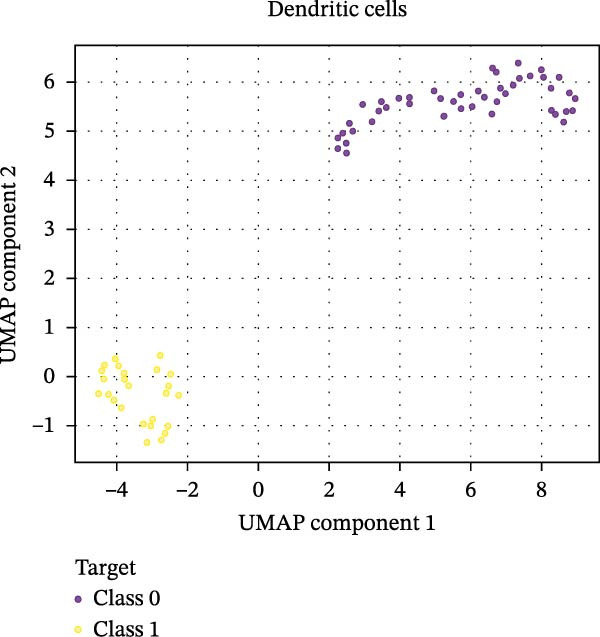
(M)

**Figure:**
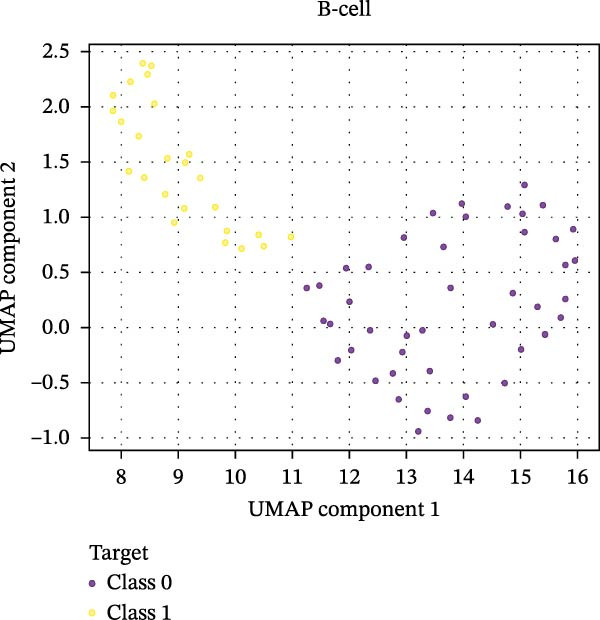
(N)

Initial ML models combining immune cell and cytokine data indicated that cytokine profiles had a stronger predictive association with later aGVHD onset compared to cellular subsets, as visualized in the correlation heatmap (Figure 6B). Among cytokines, IL‐6 and IP‐10 showed the highest positive correlation (*r* = 1.00) with aGVHD, followed by TNF‐α, RANTES, IL‐12 (p70), and IFN‐γ (*r* = 0.99), and slightly lower correlations for IL‐1β, MIP‐1α, IL‐5, and MIP‐1β. In contrast, IL‐17A, IL‐4, IL‐2, and IL‐10 were negatively correlated, with IL‐10 showing the strongest inverse relationship (*r* = –0.99) (Figure 6C). The receiver operating curve (ROC) illustrating the predictive performance of the cytokines (IL‐6, IP‐10, and TNF‐α) is shown in Figure S5A–C.

The immune cell correlation map (Figure 6D–H) highlighted strong positive correlations between aGVHD and T‐cell (total, helper, and cytotoxic), NK cells, DCs, B cell, and their subsets (which subsets) (*r* = 0.96–0.59). Lower correlation was observed for cDC1 (*r* = 0.25), and no correlation was found for KIR^−^ cytotoxic NK cells. Negative correlations were noted for immunoregulatory subsets, including Th9, Th2, Tregs, regulatory NK cells, and KIR^-^ regulatory NK cells (*r* = –0.32 to −0.77).

Uniform Manifold Approximation and Projection (UMAP) visualization (Figure 6I–N) revealed distinct clustering of aGVHD and non‐aGvHD patients based on their cytokine and immune cell profiles at engraftment. Notably, cytokine‐based UMAP plots (Figure 6I) showed clearer separation, underscoring their discriminative potential. Cellular UMAPs also demonstrated group‐level differences, further supporting their role in early immune surveillance and risk stratification for aGvHD.

An important consideration for predictive modeling is the comparability of baseline characteristics between the training and validation cohorts. In our study, the two cohorts were broadly comparable with respect to key clinical variables, including age, underlying disease type, donor source, conditioning regimen, and GvHD prophylaxis. This comparability supports the generalizability of our ML models and reduces the likelihood that differences in baseline characteristics confounded the observed predictive performance. Similar baseline profiles between cohorts strengthen the validity of our findings and suggest that the models may apply to independent patient populations with similar clinical features.

### 3.8. Distinct Immune Reconstitution Associated With aGvHD and Non‐aGvHD Patients

In this study, we longitudinally assessed immune cell reconstitution in aGvHD and non‐aGvHD patients from the point of neutrophil engraftment (Day +14) through Day +180, with evaluations at Days +30, +60, +100, and +180. Our goal was to characterize distinct trajectories in immune recovery that may underlie or predict aGvHD development.

#### 3.8.1. T‐Cell Dynamics

aGvHD patients consistently exhibited significantly higher total T‐cell counts from *D* + 14 through *D* + 100 compared to non‐aGvHD patients, suggesting accelerated or dysregulated T‐cell recovery in the aGvHD cohort. However, by *D* + 180, T‐cell counts in both groups converged (Figure S6A). This trend was mirrored in both CD4^+^ and CD8^+^ subsets (Figure S6B,C): aGvHD patients had elevated levels early on, but differences diminished over time. CD4^+^ T‐cell counts rose in the non‐aGvHD group to match aGVHD levels by *D* + 180, while CD8^+^ T‐cell counts in aGvHD patients declined, aligning with non‐aGvHD levels.

#### 3.8.2. CD4^+^/CD8^+^ Ratio and Tregs

The CD4^+^/CD8^+^ ratio remained significantly lower in aGvHD patients through *D* + 100, reflecting a CD8^+^‐dominant profile. However, a progressive rise in this ratio from *D* + 100 to *D* + 180 in the aGvHD group suggests a delayed rebalancing of T‐cell subsets (Figure S6D). While no significant difference in Treg counts was observed at *D* + 14, both groups showed increased Treg levels over time. Still, aGvHD patients consistently exhibited lower Treg counts compared to non‐aGvHD patients, with minimal increase between *D* + 100 and *D* + 180, unlike the sharp rise observed in the non‐aGvHD cohort (Figure S6E).

#### 3.8.3. NK Cell Reconstitution

NK cells were significantly elevated in aGvHD patients from *D* + 14 to *D* + 100. These levels plateaued and declined by *D* + 180. In contrast, non‐aGvHD patients demonstrated a gradual increase, with a marked rise from *D* + 100 to *D* + 180, leading to convergence of NK cell counts between groups by the end of the study period (Figure S6F).

#### 3.8.4. DCs

DC counts were initially comparable but increased more rapidly in aGvHD patients from *D* + 30 to *D* + 60, followed by a decline. Non‐aGVHD patients showed a steadier, less pronounced trajectory. Overall, DC levels remained higher in the aGVHD group throughout follow‐up (Figure S6G).

#### 3.8.5. B Cell Recovery

B cell counts were initially similar between groups. However, non‐aGvHD patients showed continuous, robust expansion from *D* + 14 to *D* + 180. In contrast, aGvHD patients experienced a delayed and more modest increase, particularly between *D* + 60 and *D* + 180, resulting in persistently lower B cell levels in this group (Figure S6H).

Together, these longitudinal data highlight distinct patterns of immune reconstitution in patients who develop aGvHD, particularly in T‐cell, NK cell, and DC compartments, suggesting that early immune dynamics may predict disease onset and progression.

## 4. Discussion

This study provides an in‐depth characterization of early immune reconstitution at the time of neutrophil engraftment and its association with subsequent development of aGvHD in Allo‐HSCT recipients, as previously shared in our preprint [4]. Our findings highlight distinct immunological signatures that precede aGvHD, implicating early dysregulated immune recovery as a driver of disease pathogenesis and offering potential biomarkers for preemptive risk stratification.

Neutrophil engraftment is a logical time point for identifying biomarkers for aGvHD because it marks the early phase of immune reconstitution following Allo‐HSCT. This period precedes the typical onset of aGvHD symptoms, offering a window for early risk prediction. As donor immune cells begin to interact with host tissues, initial immune dysregulation may become detectable. Sampling at this clinically standard milestone is both biologically relevant and logistically practical, enabling timely intervention before overt disease develops.

T‐cell dysregulation emerged as a prominent hallmark in patients who later developed aGvHD. Despite global lymphopenia during engraftment, these patients exhibited significantly elevated total T‐cell counts, particularly in both Th and Tc subsets, and reduced Tregs, corroborating earlier findings that highlighted the role of T‐cell expansion in the pathophysiology of aGvHD [7]. The expansion of effector and memory T‐cell subsets further underscores the presence of antigen‐experienced, potentially alloreactive populations. Elevated Th1, Th17, and Th22 cells, coupled with reduced Tregs, Th2 and Th9 cells, reflect a polarized proinflammatory T helper cell profile [8–11]. The dynamic imbalance between increased proinflammatory Th1/Th17 cells and decreased immunoregulatory Th2 cells and Tregs underpins the pathogenesis of aGvHD, highlighting the critical role of Tregs in restraining effector T‐cell activity and maintaining immune tolerance after Allo‐HSCT [12, 13].

NK cells, though traditionally considered protective against aGvHD, also demonstrated altered dynamics. The aGvHD cohort showed a marked expansion of cytotoxic NK cells and reduced regulatory NK cells, particularly KIR^−^ subtype, indicating not just numerical imbalance but functional skewing. The elevated proportion of KIR^+^ cytotoxic NK cells and reduced KIR^−^/KIR^+^ ratios further suggest the loss of inhibitory checkpoints and sustaining inflammatory responses and enhancing tissue damage in aGvHD through direct cytotoxic effects on host tissues [14].

DCs, particularly cDC2, were significantly elevated in patients who progressed to aGvHD. Given their potent antigen‐presenting capacity and ability to drive Th1/Th17 responses, the predominance of cDC2 may act as a key upstream trigger for the observed T‐cell activation. The expansion of both pDCs and cDCs reinforces the hypothesis of APC‐driven T‐cell priming as an early initiating event in GvHD [15, 16].

An unexpected finding in our study was the elevation of total B cell and their subsets in patients who later developed aGvHD, as B cell dysregulation is more commonly associated with chronic rather than acute GvHD [17, 18]. However, recent reports suggest that B cells may also contribute to early alloreactivity after Allo‐HSCT [19–21]. Activated B cells can function as antigen‐presenting cells through MHC class II and costimulatory molecule expression, thereby enhancing alloreactive T‐cell priming. They are also capable of producing proinflammatory cytokines such as IL‐6, TNF‐α, and IL‐1β, which can amplify T‐cell activation and tissue inflammation during early immune reconstitution [21–23]. In our cohort, this increase in B cell counts was observed only at the early engraftment time point (Day + 14), after which B cell numbers in the aGvHD group were consistently lower than in patients who did not develop aGvHD from Day + 30 through Day + 100. This pattern suggests that the early rise in B cells may represent a transient, lymphopenia‐driven reactive expansion rather than a sustained pathogenic signature. These observations highlight the nuanced and temporally dynamic role of B cells in shaping posttransplant immune responses and warrant further investigation in larger, stratified cohorts.

Previous reports have shown that aGvHD is characterized by a distinct proinflammatory signature [2, 24], which was also reflected in our study cohort. Specifically, patients who developed aGvHD, irrespective of grade, demonstrated elevated levels of IL‐6, TNF‐α, IP‐10, and IFN‐γ, coupled with suppressed regulatory cytokines such as IL‐10 and IL‐4. Notably, none of the patients developed grade I aGvHD, and among the 25 patients with aGvHD, 7 had grade II, while 18 had grades III–IV. Despite the smaller number of grade II cases, the consistent elevation of IL‐6 and TNF‐α across all observed aGvHD grades suggests that these cytokines serve as general markers of aGvHD onset rather than being restricted to severe manifestations. ML models corroborated this, identifying cytokine levels as stronger predictive markers than cellular parameters, with IL‐6, IP‐10, and TNF‐α emerging as top candidates. Together, these findings reflect a state of heightened immune activation at engraftment that may facilitate the development and progression of aGvHD, highlighting the potential utility of early cytokine monitoring for risk stratification and mechanistic insights into disease pathogenesis.

Longitudinal analyses further confirmed divergent immune reconstitution trajectories in aGvHD vs. non‐aGvHD patients. Early hyper‐reconstitution of T and NK cells, sustained DC elevation, and delayed B cell recovery characterized the aGvHD cohort and these differences converged by *D* + 180, but the prolonged CD4^+^/CD8^+^ imbalance and Treg deficit suggest lasting impacts on immune competence underscoring the need for therapeutic strategies that focus on enhancing Tregs function and/or expansion in the posttransplant period as the commonly used immunosuppressive therapies that may hinder recovery of Tregs [25, 26].

In this study, ML‐based integration of high‐dimensional immune profiling data at the time of neutrophil engraftment unveiled a set of early predictive biomarkers for aGvHD. Notably, proinflammatory cytokines such as IL‐6 and IP‐10 showed perfect correlation (*r* = 1.00) with aGvHD, underscoring their potential as robust predictors. These cytokines are known mediators of T‐cell activation, trafficking, and effector function—all of which are central to aGvHD pathogenesis. Similarly, TNF‐α, IFN‐γ, RANTES, and IL‐12 (p70), which also showed strong positive correlations, have been previously implicated in tissue injury and the amplification of alloimmune responses. In contrast, IL‐10, IL‐4, and IL‐2 cytokines were involved in immune regulation and tolerance that were negatively correlated with aGvHD, suggesting a deficiency in counter‐regulatory signals at engraftment in patients predisposed to disease.

These observations were corroborated by UMAP visualization, where cytokine‐based clustering more clearly distinguished patients who developed aGvHD from those who did not. This pattern suggests that soluble inflammatory mediators, more so than immune cell subset frequencies alone, may reflect the immunological tone most indicative of future pathology. Such a finding is clinically relevant, as cytokine profiling is more amenable to rapid, scalable, and cost‐effective testing in clinical laboratories compared to extensive flow cytometry panels.

These findings collectively suggest that a cytokine‐centric immune signature, particularly one highlighting the imbalance between inflammatory and regulatory mediators, is highly predictive of aGvHD. Importantly, the strong correlations seen in IL‐6, IP‐10, and TNF‐α suggest these may be prioritized as candidate biomarkers for clinical monitoring or even therapeutic targeting. The availability of cytokine inhibitors (e.g., IL‐6R blockade with tocilizumab and TNF‐α blockade with etanercept or infliximab) may also inform early, biologically guided interventions in high‐risk patients.

Unlike a previous study, which primarily focused on clustering T‐cell subsets using dynamic time warping to assess heterogeneity in immune reconstitution and its association with clinical outcomes such as aGvHD [27]. Our study expands the analysis by including multiple immune cell lineages and cytokine milieu at a critical clinical time point. Moreover, we employ a broader ML framework that integrates both cellular and soluble immune parameters, aiming to improve the prediction of aGvHD risk and immune recovery trajectory more holistically.

This study has several limitations that should be considered when interpreting the findings. First, although strong statistical associations were observed, the relatively small cohort size limits the generalizability of the results and may contribute to overestimation of predictive performance. In particular, the observation of perfect discriminatory ability (AUC = 1.00) for multiple cytokines raises concerns regarding potential model overfitting and cohort‐specific effects. Accordingly, these findings should be interpreted cautiously. The analyses were exploratory in nature and aimed at identifying candidate immune and cytokine features of potential relevance in aGvHD rather than establishing definitive diagnostic or prognostic biomarkers. Independent external validation in larger, multicenter cohorts is essential before any clinical application can be considered.

Second, the patient cohort was heterogeneous, encompassing a broad range of disease indications (malignant and nonmalignant), donor sources, conditioning intensities, and GvHD prophylaxis strategies (ATG‐ or PTCy‐based). While this heterogeneity reflects real‐world transplant practice at our center, it may also introduce clinical and biological confounders, as these variables independently influence the risk of acute GvHD and early immune reconstitution. For example, donor type, conditioning intensity, and GvHD prophylaxis are known to markedly affect immune subset distribution and kinetics; notably, PTCy selectively targets proliferating alloreactive T‐cells while relatively sparing regulatory T‐cells, potentially shaping the immune signatures detected in our analysis. Studies in more homogeneous cohorts and stratified prospective designs will be required to further validate and refine these observations.

Third, the present study focused primarily on phenotypic immune profiling. The absence of functional immune assays limits the mechanistic interpretation of the observed immune alterations and their direct contribution to GvHD pathophysiology. Incorporation of functional assessments in future studies would provide deeper insight into immune dysregulation and enhance biological interpretation.

Additionally, although ROC analyses were employed to assess discriminatory potential, the study was not powered to define or validate clinically actionable biomarker cutoff thresholds. Establishing such thresholds will require larger cohorts, longitudinal sampling, and outcome‐driven optimization. Consequently, the lack of validated cutoffs limits the immediate translational applicability of these findings.

Finally, the cohort predominantly comprised patients with moderate to severe aGvHD (grade II–IV), and therefore, the findings may not be generalizable to patients with mild (grade I) disease. Extrapolation to early or low‐grade GvHD should thus be approached with caution, and dedicated studies focusing on the full spectrum of disease severity are warranted.

Additionally, the impact that NRM and cGvHD could have on the prognosis must be considered while interpreting our findings. Although this study focused mostly on early immune reconstitution and acute GvHD, we acknowledge that both of these factors are critical determinants of long‐term outcomes after Allo‐HSCT. Early immune signatures predictive of aGvHD may also contribute to susceptibility to cGvHD or risk of NRM, but these outcomes could not be comprehensively assessed in our current cohort and follow‐up duration. Longer follow‐up and larger patient numbers are necessary in future studies to assess how early immune perturbations relate not only to acute complications but also to long‐term survival, NRM, and development of cGvHD. Their recognition is important for a holistic understanding of posttransplant immune recovery and prognosis.

## 5. Conclusion

Our comprehensive analysis underscores the critical immunological profiles that emerge at the time of neutrophil engraftment and their implications for the prediction of aGvHD development. In the correlation of cellular and cytokine responses with existing literature, we emphasize the complex interplay between different immune subsets and the necessity for refined monitoring and potential therapeutic interventions aimed at optimizing graft acceptance and reducing aGvHD incidents.

Together, these data support a model in which aberrant immune reconstitution, marked by T‐cell skewing, proinflammatory cytokine dominance, and APC expansion, predisposes patients to aGvHD. Our findings underscore the potential utility of early immune monitoring, not only for predicting aGvHD risk but also for guiding personalized interventions such as prophylactic immunomodulation or early therapy escalation.

**Nomenclature**
Allo‐HSCT: Allogeneic hematopoietic stem cell transplantation
aGvHD: Acute graft‐versus‐host‐disease
ANC: Absolute neutrophil count
ML: Machine learning
ELISA: Enzyme‐linked immunosorbent assay
PB: Peripheral blood
Tregs: Regulatory helper T‐cell
NK: Natural killer
IFN‐γ: Interferon‐gamma
IL‐1β: Interleukin‐1beta
IP‐10: Interferon gamma‐induced protein 10
TNF‐α: Tumor necrosis factor‐alpha
IL‐17α: Interleukin‐17 alpha
IL‐12p70: Interleukin‐12p70
MIP‐1α: Macrophage inflammatory protein 1‐alpha
MIP‐1β: Macrophage inflammatory protein 1‐beta
RANTES: Regulated upon activation, normal T‐cell expressed and secreted
SVC: Support vector classifier
RBF: Radial basis function
AML: Acute myeloid leukemia
ALL: Acute lymphoblastic leukemia
CML: Chronic myeloid leukemia
MDS: Myelodysplastic syndrome
CLL: Chronic lymphocytic leukemia
MSD: Myelodysplastic syndromes
PTCy: Posttransplant cyclophosphamide
ATG: Antithymocyte globulin
TBI: Total body irradiation
MTX: Methotrexate
CNI: Calcineurin inhibitors
MMF: Mycophenolate mofetil.

## Author Contributions

Mohini Mendiratta performed the experiments, acquired and analyzed data, interpreted the results, and wrote the manuscript. Praful Pandey, Shobhit Pandey, Sandeep Rai, Hridayesh Prakash, Shuvadeep Ganguly, Archana Sasi, Ritu Gupta, Prabhat Singh Malik, Raja Pramanik, Sachin Kumar, Baibaswata Nayak, and Riyaz Ahmed Mir contributed to data interpretation and analysis. Meenakshi Mendiratta performed experiments. Sameer Bakhshi, Deepam Pushpam, Mukul Aggarwal, Aditya Kumar Gupta, Rishi Dhawan, Tulika Seth, and Manoranjan Mahapatra provided patient samples and their clinical details. Ranjit Kumar Sahoo provided funding and resources, conceptualized the study, designed and supervised the experiments, analyzed data, and edited the manuscript.

## Funding

The study has been supported by the Indian Council of Medical Research, New Delhi, India (Grant 3/2/2/55/2022‐NCD‐III).

## Disclosure

All authors critically reviewed and approved the final version of the manuscript.

## Conflicts of Interest

The authors declare no conflicts of interest.

## Supporting Information

Additional supporting information can be found online in the Supporting Information section.

## Supporting information


**Supporting Information** Figure S1: Dot plots illustrate the gating strategy for (A) T‐cell, subtypes of T‐cell, Th cell, Tc cell, and Tregs. (B) Subtypes of effector memory Th cell. Th, helper T‐cell, Tc, cytotoxic T‐cell; Treg, regulatory helper T‐cell. Figure S2: Dot plots illustrate the gating strategy for (A) NK cell and their subtypes. (B) Dendritic cells and their subtypes. NK, natural killer; KIR, killer‐cell immunoglobulin‐like receptor; DC, dendritic cell. Figure S3: Dot plots illustrate the gating strategy for B cell and their subtypes. SM, switched memory; USM, unswitched memory; DNSM, double negative switched memory. Figure S4: B cell and their subtypes at the neutrophil engraftment of the patients who either did or did not develop aGvHD in the later phase and healthy control using flow cytometry. The scatter plot represents the absolute count (cells/μL) of (A) CD19+ B cell. (B) IgD+ CD27− naïve B cell. (C) IgD+ CD27+ USM B cell. (D) IgD− CD27+ SM B cell. (E) IgD−CD27−DNSM B cell. Data are presented as the median with interquartile range for 20 healthy controls and 70 patients (aGvHD = 25; non‐aGvHD = 45). Statistical analysis: Mann–Whitney test;  ^∗∗∗∗^≤0.0001. USM, unswitched memory; SM, switched memory; DNSM, double negative switched memory; aGvHD, acute graft‐versus‐host disease. Figure S5: Receiver operating characteristic (ROC) analysis of cytokine levels for the prediction of acute GVHD. Plots depict ROC curves for (A) IL‐6, (B) IP‐10, and (C) TNF‐α. All three cytokines exhibited complete separation between aGVHD and non‐GVHD patients, reflected by an AUC of 1.00 for each marker. IL‐6, interleukin; IP‐10, interferon gamma‐induced protein 10; TNF‐α, tumor necrosis factor. Figure S6: Kinetics of immune reconstitution of aGvHD and non‐aGvHD patients. The line graphs represent the absolute count (cells/μL) from D+14 to D+180 of (A) CD3+ T‐cell. (B) CD3+ CD4+ T‐cell. (C) CD3+ CD8+ T‐cell. (D). CD4+/CD8+ T‐cell ratio. (E) CD3+ CD4+ CD25+ FOXP3+ Tregs. (F) CD3− CD56+ NK cell. (G) CD11c+ HLA‐DR+ dendritic cells. (H) CD19+ B cell. Data are presented as the median with interquartile range for aGVHD patients (*n* = 25) and non‐aGvHD patients (*n* = 45). Statistical analysis: Mann–Whitney test;  ^∗^≤0.s05;  ^∗∗^≤0.01;  ^∗∗∗^≤0.001;  ^∗∗∗∗^≤0.0001. NK, natural killer; Tregs, regulatory helper T‐cell; aGvHD, acute graft‐versus‐host disease.

## Data Availability

The data that support the findings of this study will be made available from the corresponding author upon reasonable request.
