# Supplementary material for: Cytokine Signatures Outperform Immune Subsets in Machine Learning Models for Predicting Acute Graft‐Versus‐Host Disease at Neutrophil Engraftment
Source: J Immunol Res. 2026 Feb 23;2026:1066614. doi: 10.1155/jimr/1066614 (PMC13140371; doi:10.1155/jimr/1066614)
Supplement: Supplementary file 1 — Supporting Information Figure S1: Dot plots illustrate the gating strategy for (A) T‐cell, subtypes of T‐cell, Th cell, Tc cell, and Tregs. (B) Subtypes of effector memory Th cell. Th, helper T‐cell, Tc, cytotoxic T‐cell; Treg, regulatory helper T‐cell. Figure S2: Dot plots illustrate the gating strategy for (A) NK cell and their subtypes. (B) Dendritic cells and their subtypes. NK, natural killer; KIR, killer‐cell immunoglobulin‐like receptor; DC, dendritic cell. Figure S3: Dot plots illustrate the gating strategy for B cell and their subtypes. SM, switched memory; USM, unswitched memory; DNSM, double negative switched memory. Figure S4: B cell and their subtypes at the neutrophil engraftment of the patients who either did or did not develop aGvHD in the later phase and healthy control using flow cytometry. The scatter plot represents the absolute count (cells/μL) of (A) CD19+ B cell. (B) IgD+ CD27− naïve B cell. (C) IgD+ CD27+ USM B cell. (D) IgD− CD27+ SM B cell. (E) IgD−CD27−DNSM B cell. Data are presented as the median with interquartile range for 20 healthy controls and 70 patients (aGvHD = 25; non‐aGvHD = 45). Statistical analysis: Mann–Whitney test; ∗∗∗∗≤0.0001. USM, unswitched memory; SM, switched memory; DNSM, double negative switched memory; aGvHD, acute graft‐versus‐host disease. Figure S5: Receiver operating characteristic (ROC) analysis of cytokine levels for the prediction of acute GVHD. Plots depict ROC curves for (A) IL‐6, (B) IP‐10, and (C) TNF‐α. All three cytokines exhibited complete separation between aGVHD and non‐GVHD patients, reflected by an AUC of 1.00 for each marker. IL‐6, interleukin; IP‐10, interferon gamma‐induced protein 10; TNF‐α, tumor necrosis factor. Figure S6: Kinetics of immune reconstitution of aGvHD and non‐aGvHD patients. The line graphs represent the absolute count (cells/μL) from D+14 to D+180 of (A) CD3+ T‐cell. (B) CD3+ CD4+ T‐cell. (C) CD3+ CD8+ T‐cell. (D). CD4+/CD8+ T‐cell ratio. (E) CD3+ CD4+ CD25+ FOXP3+ Tregs. (F) C [file JIMR-2026-1066614-s001.docx]

**Supplementary figures**

**Figure S1**

**
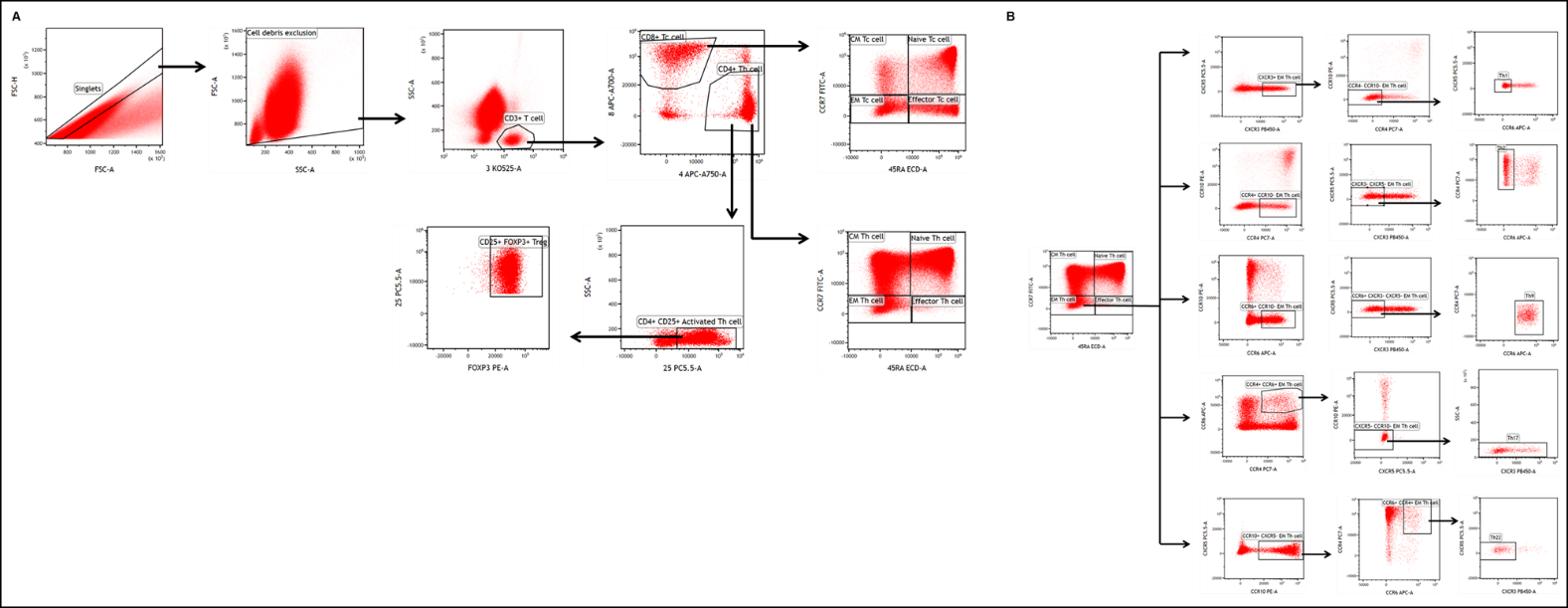
**

**Figure S1:** *Dot plots illustrate the gating strategy for (A) T-cell, subtypes of T-cell, Th cell, Tc cell, and Tregs. (B) Subtypes of effector memory Th cell. Abbreviations: Th: Helper T-cell, Tc: Cytotoxic T-cell; Treg: Regulatory Helper T-cell*

**Figure S2**

*
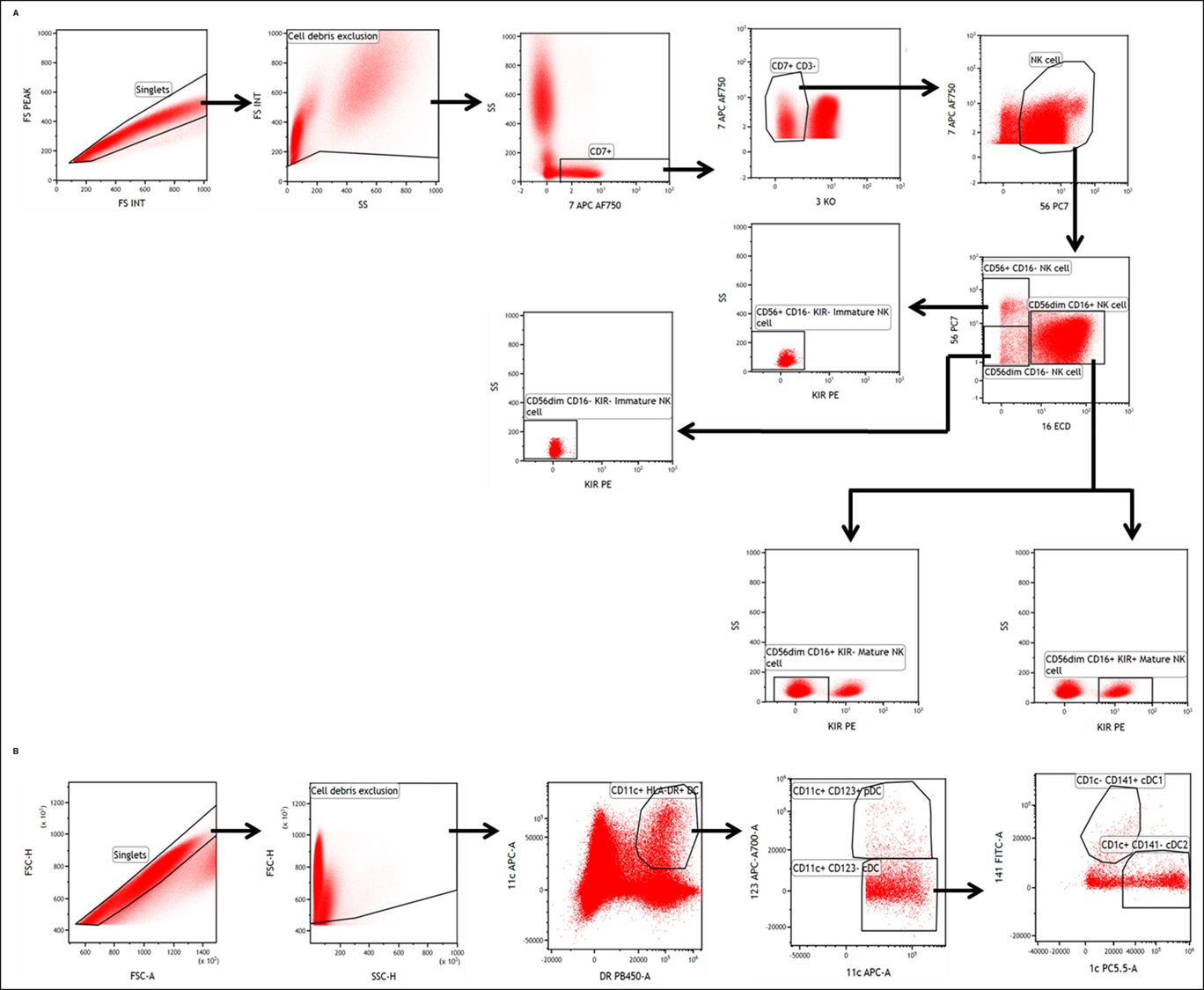
*

**Figure S2:** *Dot plots illustrate the gating strategy for (A) NK cell and their subtypes. (B) Dendritic cells and their subtypes. Abbreviations: NK: Natural Killer; KIR; Killer-cell Immunoglobulin-like Receptor; DC: Dendritic cell*

**Figure S3**

**
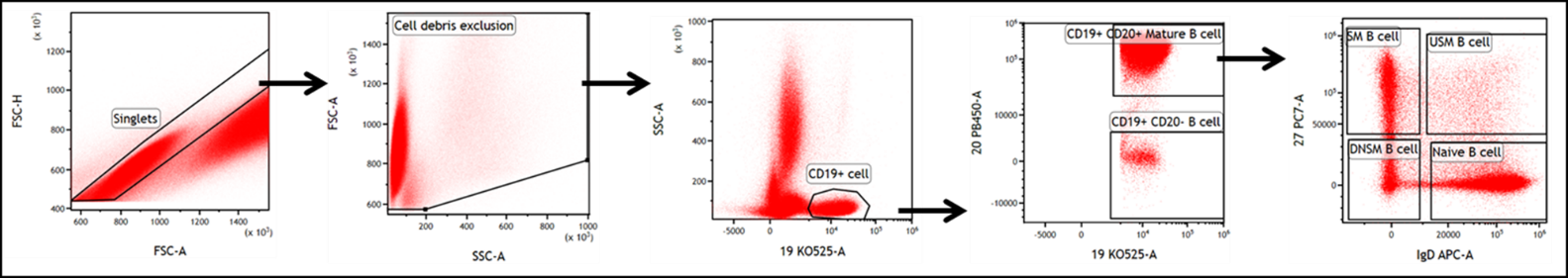
**

**Figure S3:** *Dot plots illustrate the gating strategy for B-cell and their subtypes. Abbreviations: SM: Switched Memory; USM: Unswitched Memory; DNSM: Double Negative Switched Memory*

**Figure S4**

**
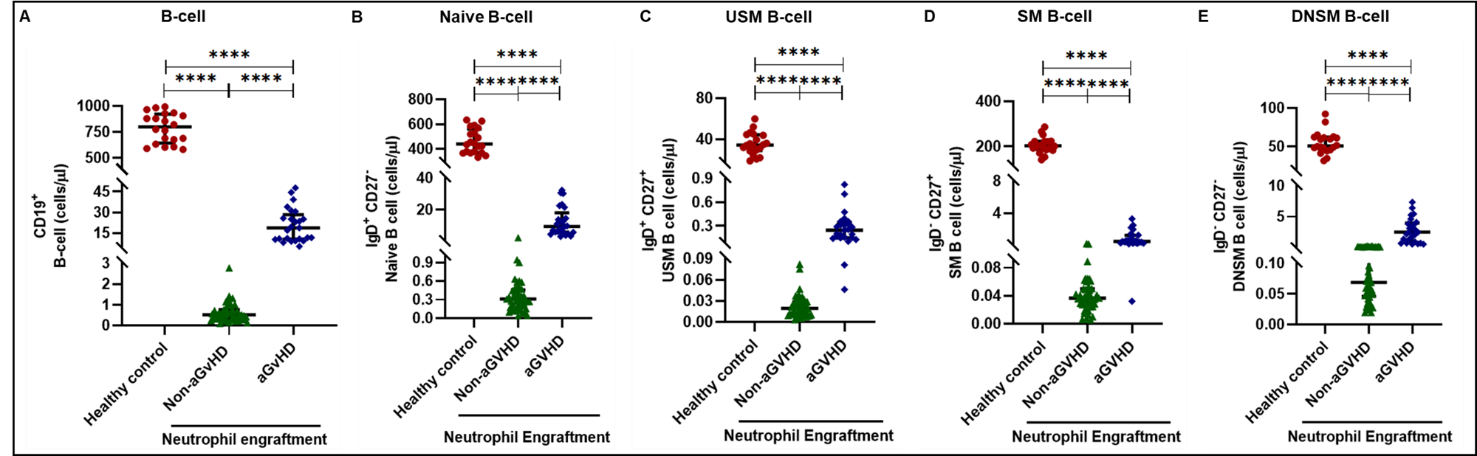
**

**Figure S4:** *B-cell and their subtypes at the neutrophil engraftment of the patients who either did or did not develop aGvHD in the later phase and healthy control using flow cytometry. The scatter plot represents the absolute count (cells/μl) of (A) CD19^+^ B-cell. (B) IgD^+^ CD27^-^ Naïve B-cell. (C) IgD^+^ CD27^+^ USM B-cell. (D) IgD^-^ CD27^+^ SM B-cell. (E) IgD^-^ CD27^-^ DNSM B-cell. Data are presented as the median with interquartile range for 20 healthy control and 70 patients (aGvHD = 25; non-aGvHD = 45). Statistical analysis: Mann-Whitney Test; ****≤0.0001. Abbreviations: USM: Unswitched Memory; SM: Switched Memory; DNSM: Double Negative Switched Memory; aGvHD: Acute Graft-versus-Host-Disease*

*
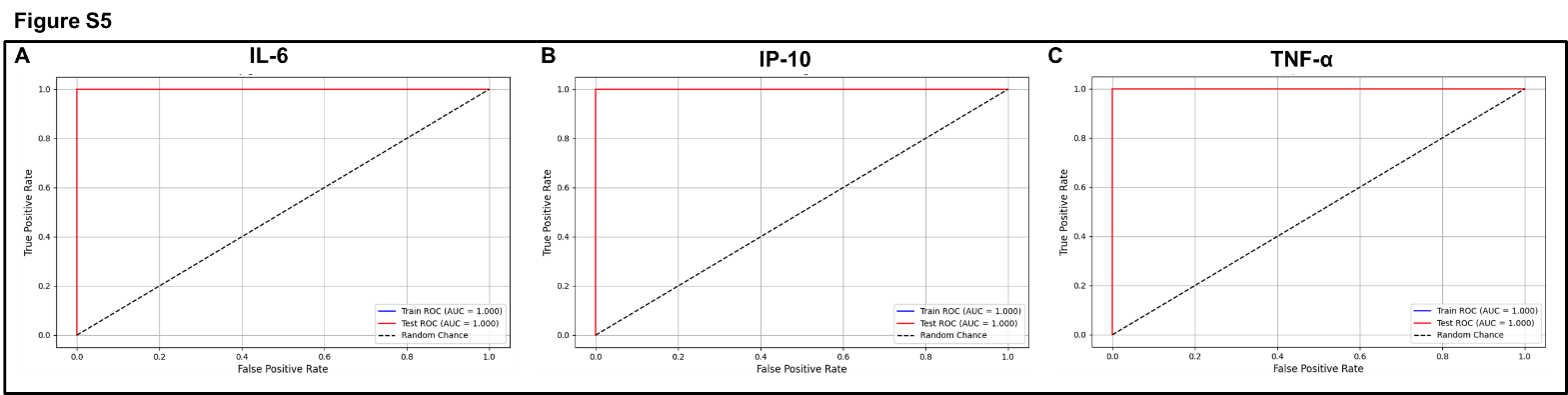
*

**Figure S5:** *Receiver operating characteristic* (*ROC) analysis of cytokine levels for the prediction of acute GVHD. Plots depict ROC curves for (A) IL-6, (B) IP-10, and (C) TNF-α. All three cytokines exhibited complete separation between aGVHD and non-GVHD patients, reflected by an AUC of 1.00 for each marker.* *Abbreviations: IL-6: Interleukin; IP-10: Interferon gamma-induced protein 10; TNF-α: Tumor Necrosis Factor*

**Figure S6**

**
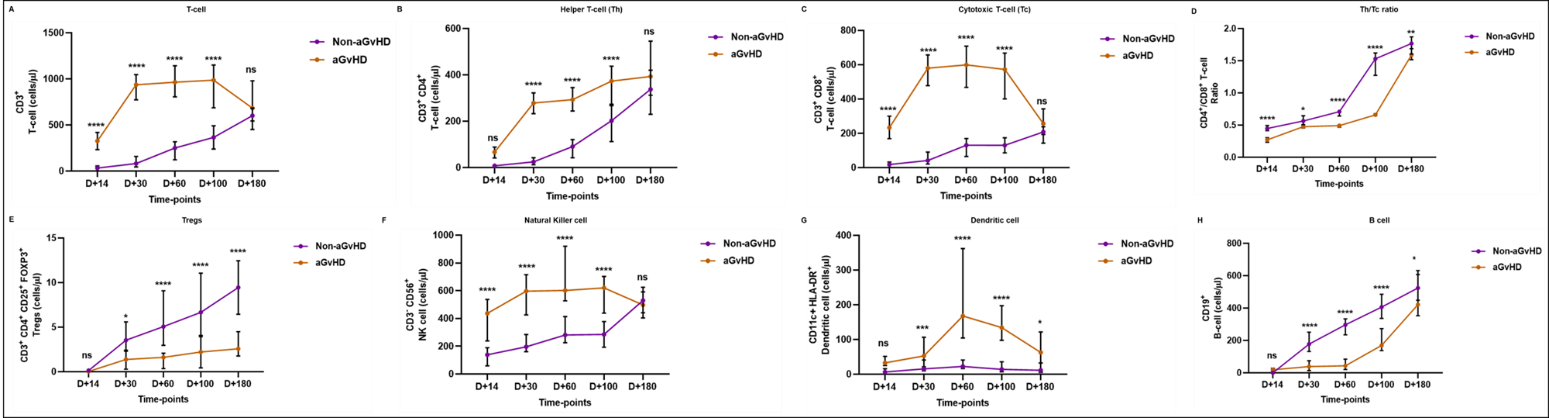
**

**Figure S6:** *Kinetics of immune reconstitution of aGvHD and non-aGvHD patients. The line graphs represent the absolute count (cells/μl) from D+14 to D+180 of (A) CD3^+^ T-cell. (B) CD3^+^ CD4^+^ T-cell. (C) CD3^+^ CD8^+^ T-cell. (D). CD4^+^/CD8^+^T-cell ratio. (E) CD3^+^ CD4^+^ CD25^+^ FOXP3^+^ Tregs. (F) CD3^-^ CD56^+^ NK cell. (G) CD11c^+^ HLA-DR^+^ Dendritic cells. (H) CD19^+^ B-cell. Data are presented as the median with interquartile range for aGVHD patients (n=25) and non-aGvHD patients (n=45). Statistical analysis: Mann-Whitney Test; *≤0.s05; **≤0.01; ***≤0.001; ****≤0.0001. Abbreviations: NK: Natural Killer; Tregs: Regulatory Helper T-cell; aGvHD: Acute Graft-versus-Host-Disease*
